# NIR-715 photodynamic therapy induces immunogenic cancer cell death by enhancing the endoplasmic reticulum stress response

**DOI:** 10.1038/s41419-024-07283-4

**Published:** 2024-12-18

**Authors:** Zhen-Yuan Zheng, Wan Lin, Jia-Wan Su, Qing-Feng Huang, Cong Zhang, Wen-Xing Pan, En-Min Li, He-Feng Zhang, Li-Yan Xu

**Affiliations:** 1 Department of Oncobiology, Department of Basic Medical Sciences, Shantou University Medical College, Shantou, Guangdong PR China; 2 The Key Laboratory of Molecular Biology for High Cancer Incidence Coastal Chaoshan Area, Cancer Research Center, Shantou University Medical College, Shantou, 515041 Guangdong P. R. China; 3 Chaoshan Branch of State Key Laboratory for Esophageal Cancer Prevention and Treatment, Shantou, China; 4 Department of Chemistry and Key Laboratory for Preparation and Application of Ordered Structural Materials of Guangdong Province, Shantou University, Science Building, 243 Daxue Road, Shantou, 515063 Guangdong PR China; 5 The Key Laboratory of Molecular Biology for High Cancer Incidence Coastal Chaoshan Area, Department of Biochemistry and Molecular Biology, Shantou University Medical College, Shantou, 515041 Guangdong China

**Keywords:** Cell death and immune response, Drug delivery, Cancer immunotherapy

## Abstract

Effectively interfering with endoplasmic reticulum (ER) function in tumor cells and simultaneously activating an anti-tumor immune microenvironment to attack the tumor cells are promising strategies for cancer treatment. However, precise ER-stress induction is still a huge challenge. In this study, we synthesized a near-infrared (NIR) probe, NIR-715, which induces tumor cell death and inhibits tumor growth without causing apparent side effects. NIR-715 triggers severe ER stress and immunogenic cell death (ICD) after visible light exposure. NIR-715 induced ICD-associated HMGB1 release in vitro and anti-tumor immune responses, including increased cytotoxic T lymphocyte (GZMB^+^ CD8^+^ T cell) infiltration and decreased numbers of exhausted T lymphocytes (PD-L1^+^ CD8^+^ T cell). These findings suggest that NIR-715 may be a novel agent for “cold” tumor photodynamic therapy (PDT).

**Figure:**
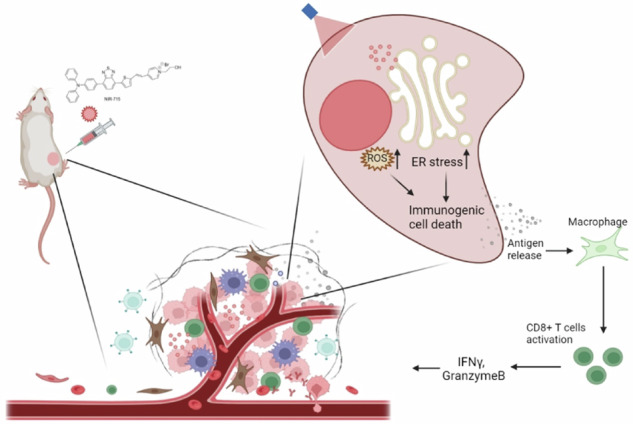
Schematic illustration of NIR-715 photodynamic therapy for visible light-triggered, endoplasmic reticulum-targeting antitumor therapy.

Schematic illustration of NIR-715 photodynamic therapy for visible light-triggered, endoplasmic reticulum-targeting antitumor therapy.

## Introduction

Tumor immunotherapy with checkpoint inhibitors has been shown great promise in various malignancies, including esophageal cancer [1–3], lung cancer and melanoma [4]. However, a proportion of patients exhibit a highly immunosuppressive tumor microenvironment (TME) and remain unable to respond to immunotherapy [5]. The diversity of immune evasion mechanisms is a key challenge in converting non-responsive, Gastineau DA, Katsanis E, Johnson BD, [6]. Hot tumors, characterized by T cells infiltration, are termed characterized by T cells ng J, Huang D, Saw PE, Song E. Turning cold tumors hot: from molecular mechanisms to clinical applications. Trends Immunol. 2022;43(7patient cohorts. Non-responders to ICB therapies often exhibit a uang D, Saw PE, Song E. Turning cold tumors hot: from molecular mechanisms to clinical applications. Trends Immunol. 2022;4ntially allow non-responders to benefit from ICB immunotherapy. The efficacy of ICB largely depends on the number of tumor-infiltrating lymphocytes (TILs) and neoantigen load [7, 8]. Thus, strategies to induce neoantigen release and TIL recruitment in the TME may enhance tumor immunogenicity and response to ICB [9, 10]. Multiple therapeutic modalities, such as chemotherapy, radiotherapy, and photodynamic therapy (PDT) are available for inducing immunogenic cell death (ICD). ICD can generate damage-associated molecular patterns (DAMPs), which may help to convert an immunologically “cold” tumor to a “hot” tumor and elicit immune responses [11–13].

The endoplasmic reticulum (ER) is a central organelle where secreted and transmembrane proteins are synthesized and processed. ER stress is highly activated and favors tumor cell survival in malignant tumors [14]. However, sustained or severe ER stress can induce tumor cell death [15]. An increasing number of studies have highlighted the relation between ER stress, reactive oxygen species (ROS) production and induction of ICD [16]. Several chemotherapeutic compounds, such as doxorubicin, oxaliplatin and mitoxantrone, as well as radiotherapy, can stimulate ICD through non-associated targets of ER and DAMP release [17–19]. However, it is more effective to trigger ICD through targeting the ER directly and inducing ROS-based ER stress [19]. It has been reported that hypericin-based PDT could target ER and induce major DAMP release, thus resulting in high levels of ICD [20, 21].

Developing bio-probes capable of PDT is attracting more and more attention [22, 23]. PDT has shown promising results in the treatment of various malignancies, including lung, esophageal, bladder, and cervical cancers [24–26]. It has also been effective in the management of premalignant conditions, such as dysplasia and squamous cell carcinoma. In typical PDT, a photosensitizer is excited and further excites a stable triplet oxygen to a reactive single state or other reactive species that can kill cells or microorganisms. However, most photosensitizers lack the ability to cause sufficient ER stress under light excitation [27].

Here, we have synthesized and characterized an NIR probe, NIR-715, which triggers ER stress and ICD after visible light exposure. NIR-715 promotes DAMP released and CD8^+^ cytotoxic T lymphocyte (CTL) intratumor infiltration to exert a potent antitumor effect.

## Results

### Characterizations of NIR-715

**Fig. 1:**
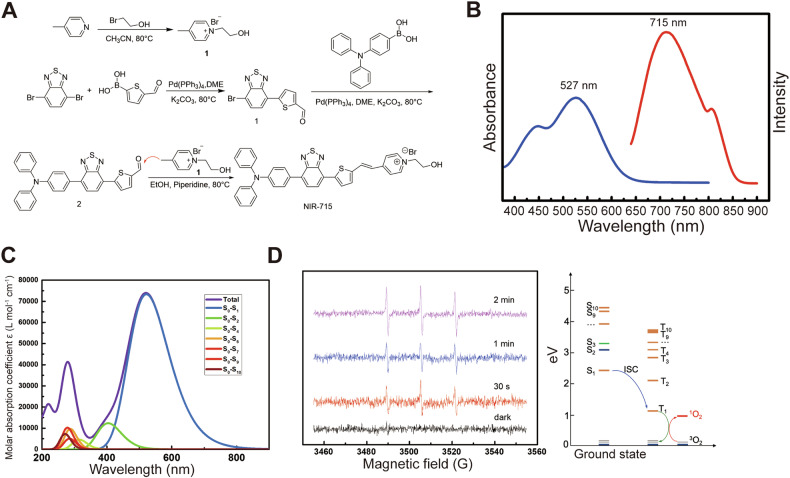
Characterization of NIR-715. **A** Synthesis of NIR-715. **B** Absorption and fluorescence of NIR-715. **C** Calculated transition contributions. **D** Generation of singlet oxygen by NIR-715 monitored by EPR spectra and the mechanism revealed by TD-DFT computation.

The synthesis of NIR-715 is shown in Fig. 1A. Intermediate 1 was synthesized by using a Suzuki coupling reaction between 4,7-dibromobenzo[c] [1,2,5] thiadiazole and (5-formylthiophen-2-yl) boronic acid in the presence of tetrakis (triphenylphosphine) palladium as catalyst followed by coupling with (4-(diphenylamino)phenyl)boronic acid under the same conditions. The resulting intermediate 2 was coupled with pre-synthesized compound 3 or 4 to give NIR-715. In the probe, triphenylamino and thiophene groups serve as electron donors and benzo[c] [1,2,5] thiadiazole and pyridinium groups serve as electron acceptors to form a D-A-D-A structure with the internal vinyl group as a π bridge. The NIR-715 probe showed NIR-I emissive fluorescence at *λ*_em_ = 715 nm with a subpeak at *λ*_em_ = 806 nm with max absorption at *λ*_abs_ = 526 nm and second excitation at *λ*_abs_ = 446 nm (Fig. 1B). NIR emission and large Stokes shifts (> 180 nm) conferred on the probe high penetrability and signal-to-noise ratio in bio-imaging. TD-DFT calculation was performed to disclose contributions of transitions to the absorption. It was found that main absorption peak at *λ*_abs_ = 527 nm and sub peak at *λ*_abs_ = 446 nm were contributed by the transition from s_0_ to s_1_ and s_0_ to the other excitation states with higher energies than that of s_1_ (Fig. 1C). NIR-715 showed strong solvent dependence, that is, the probe showed a much stronger fluorescence emission in solvents with low polarity, such as dichloromethane, than in polar solvents such as ethanol and DMSO. The strong non-radiative transition in high polar solvents promised enough energy for phototherapy performance (Supplementary Fig. S1–3).

NIR-715 showed weak fluorescence emission in aqueous solution, which implies possible good photothermal or photodynamic performance. EPR spectra were used to monitor the generation of singlet oxygen species in aqueous solution with an Xe lamp, by which the excellent photodynamic performance of NIR-715 was discovered. As shown in Fig. 1D, the aqueous solution of NIR-715 did not show any signal of singlet oxygen without any light. After exposure to Xe light for 30 s, singlet oxygen species were generated and increased with exposure time. The singlet oxygen promised excellent photodynamic performance. The driving force for singlet oxygen generation was from transition of NIR-715 from T1 to T0 with an energy of 1.14 eV, which was high enough to activate oxygen molecules in solution from the triplet to singlet state, which requires 0.98 eV.

### Intracellular uptake and antitumor effects of NIR-715 in vitro and in vivo

**Fig. 2:**
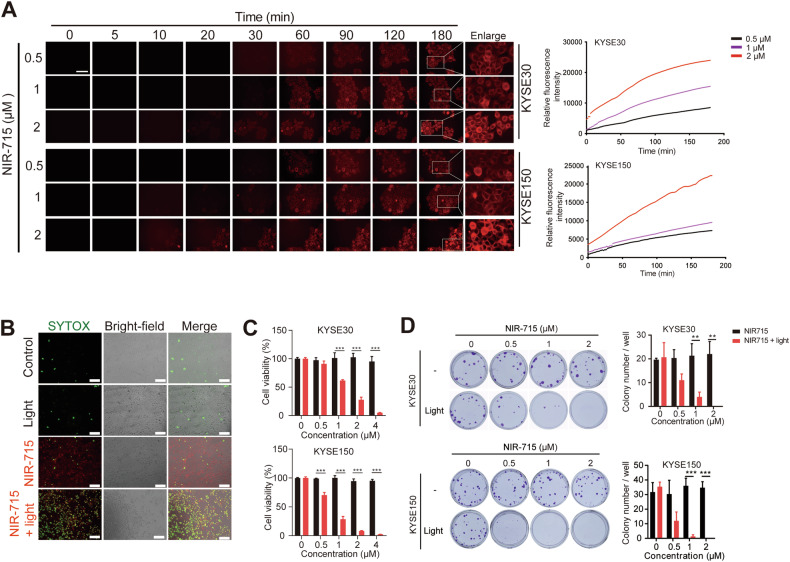
Intracellular uptake and antitumor effect of NIR-715 in vitro. **A** Uptake of NIR-715 by KYSE30 and KYSE150 cells, imaged by live-cell confocal fluorescence microscopy, at various times after NIR-715 addition. Fluorescence intensity was measured with a fluorescence microscope. Scale bar, 100 μm. **B** KYSE30 cells treated with vehicle (1% DMSO) or NIR-715 (2 μM) followed by light exposure (10 min) and SYTOX Green staining, as detected by live-cell confocal fluorescence microscopy. Scale bar, 100 μm. **C** Cell viability of KYSE30 (upper) and KYSE150 (lower) cells treated with various concentrations of NIR-715 with or without light exposure for 10 min. Cell viability was evaluated by MTS assay. **D** Colony formation assay for KYSE30 (upper) and KYSE150 (lower) cells after NIR-715 treatment. All data are presented as the mean ± SD (*n* = 4). **P < 0.01 ***P < 0.001.

To characterize the antitumor effect of NIR-715, we first investigated the effects of NIR-715 on intracellular uptake. NIR-715 fluorescence intensity in the cells gradually increased with incubation time and concentration (Fig. 2A, Supplementary Fig. S4 and Video 1). Given the stability and efficiency of photodynamic conversion of NIR-715, we examined the ability to kill esophageal cancer cells in vitro. The combination of visible light exposure and NIR-715 treatment induced cell death, whereas light exposure or NIR-715 alone had little effect on cell death (Fig. 2B and Supplementary Fig. S5). Next, we analyzed NIR-715 cytotoxicity with or without visible light exposure by using an MTS assay. Incubating KYSE30 and KYSE150 cells with NIR-715 2 h and light exposure inhibit cell proliferation obviously at NIR-715 concentrations above 1 μM (Fig. 2C). The same effect was also observed for breast cancer and melanoma cell lines (Supplementary Fig. S6A). 24 h of NIR-715 incubation without withdrawal had the same effect in multiple cell lines (Supplementary Fig. S6B). Moreover, in the absence of light exposure, NIR-715 uptake does not cause toxicity to the cells (Supplementary Fig. S7). Colony formation assays showed that NIR-715 plus light exposure decreased colony formation (Fig. 2D). These results suggest that NIR-715 has high cell uptake efficiency and antitumor ability.

**Fig. 3:**
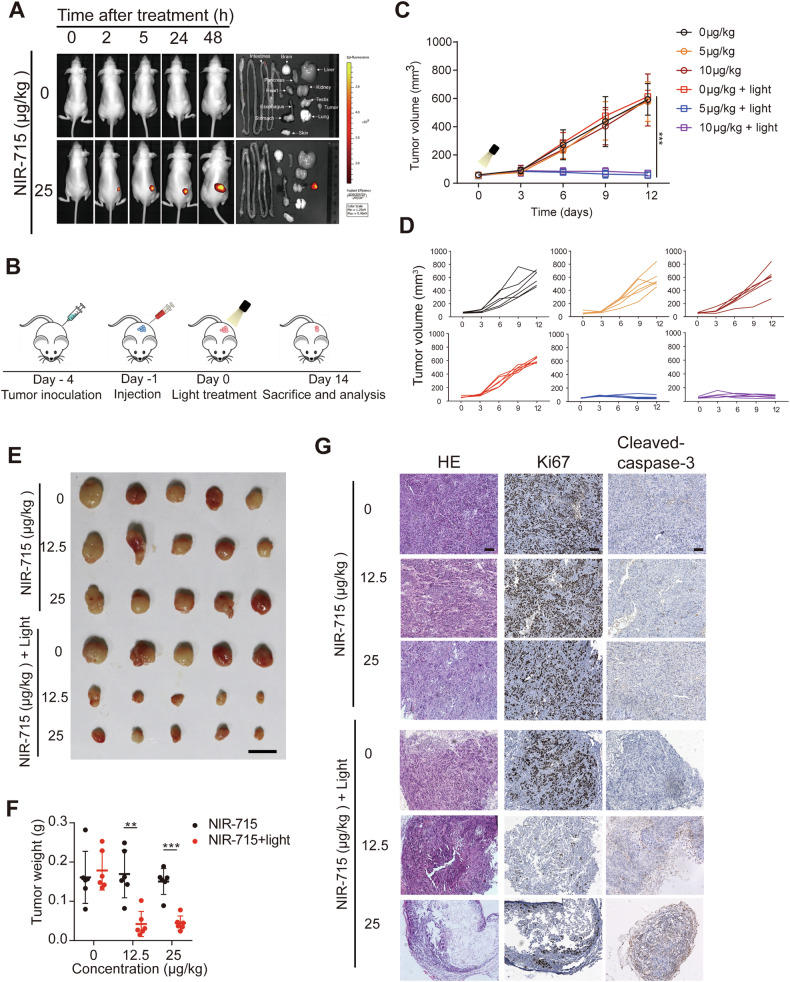
Antitumor effects of NIR-715 in tumor-bearing nude mice. **A** IVIS fluorescence imaging of KYSE30-tumor bearing BALB/c nude mice after intratumoral injection with NIR-715 or PBS control. Epi-fluorescence imaging of mouse organs (tumor, heart, liver, spleen, lung, kidney, intestines, stomach, brain). **B** Schematic diagram of KYSE30 tumor-bearing BALB/c nude mouse establishment and treatment in vivo. **C** Relative tumor volume growth curves of mice in different groups during the process of different concentrations of NIR-715 therapy. Data are presented as mean ± SEM (*n* = 5 independent samples). **D** Tumor volumes of individual mice in different groups of KYSE30 tumor-bearing nude mice. **E** Image of nude mice bearing KYSE30 tumors treated with NIR-715 with or without light exposure. Scale bar: 1 cm. **F** Tumor weight scatter diagram after different treatments (*n* = 5). **G** H&E, Ki67 and cleaved caspase-3 staining of tumor sections from xenografts on day 14 after different treatments. Scale bar, 100 μm. **P < 0.01 ***P < 0.001.

To investigate the vivo tumor retention, NIR-715 and PBS were administered intratumorally into two groups of KYSE520 cells bearing nude mice. From IVIS images, NIR-715 fluorescence was observed at 48 h (Fig. 3A). Due to its great cell uptake and stability, NIR-715 showed persistent tumor retention. Next, we used nude mice bearing KYSE30 tumors to further validate whether NIR-715 has antitumor activity in vivo. Tumor-bearing mice were treated with an intratumor injection of PBS, 12.5 μg/kg NIR-715 and 25 μg/kg NIR-715 when the tumor reached 100 mm^3^ on day 4. The next day, visible light exposure was applied to the mice for PDT (Fig. 3B). The combined NIR-715 and light exposure showed suppressed tumor growth compared to PBS or light exposure alone (Fig. 3C–F). IHC also showed decreased Ki67 and increased cleaved caspase-3 expression in xenografts upon combination treatment (Fig. 3G). These results suggest the potent antitumor efficacy of the combined NIR-715 and PDT strategy has great promise for the treatment of EC.

### NIR-715 induces ROS generation and disturbs ER morphology

**Fig. 4:**
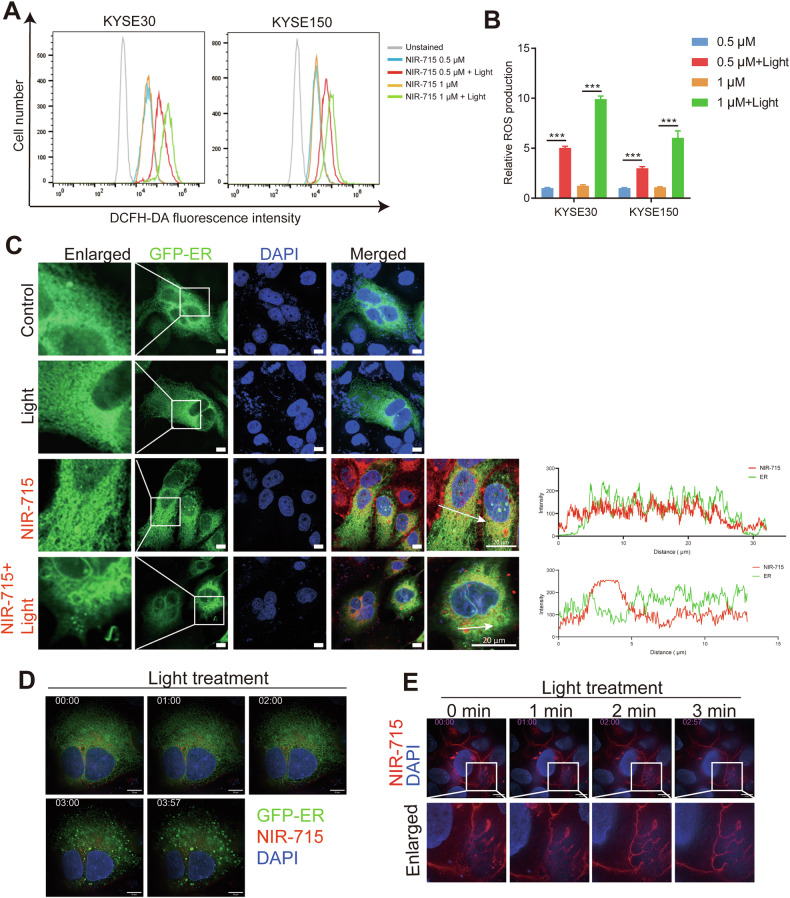
ROS generation and endoplasmic reticulum (ER) morphology disruption. **A**, **B** ROS generation was measured with DCFH-DA. KYSE30 and KYSE150 cells were treated 2 h with the indicated concentrations of NIR-715, then the cells were refed with fresh culture medium and exposure with or without light, and the DCFH-DA fluorescence intensity was measured by flow cytometry. **C** Confocal fluorescence images of ER and NIR-715 after treatment with light irritation. Green fluorescence represents ER, red fluorescence represents NIR-715. **D** Fluorescence images of ER morphology in live cells after treatment with NIR-715. **E** Fluorescence images of NIR-715 in live cells after light treatment. Scale bar: 10 μm. Error bars represent the mean ± SD of three independent experiments. ***P < 0.001.

To determine whether NIR-715 promotes tumor cell death through single oxygen species generation, intracellular ROS generation was evaluated in KYSE30 and KYSE150 cells using 2′,7′-dichlorodihydrofluorescein diacetate (DCFH-DA) as a fluorescence probe. NIR-715 induced excessive ROS generation under visible light exposure in a NIR-715 dose-dependent manner (Fig. 4A, B). To identify the intracellular effect of NIR-715, we performed a confocal fluorescence microscopy on KYSE150 cells. As shown in Fig. 4C, we observed marked disorganization of the peripheral ER network after NIR-715 plus visible light exposure in KYSE150 cells stably expressing the ER localization fluorescent reporter protein (GFP-ER). Further ultrastructural validation was obtained by high sensitivity structured illumination microscopy (HIS-SIM). After treatment with NIR-715, KYSE150-GFP-ER cells were observed by live-cell real-time super resolution microscopy (Fig. 4D, and Video 2). We also found cell membranes to bleb, burst and die after NIR-715 activation (Fig. 4E, and Video 3). These data confirm that NIR-715 induces ROS generation and disrupt ER morphology in EC cells, eventually inducing cell death.

### ER stress and ICD

**Fig. 5:**
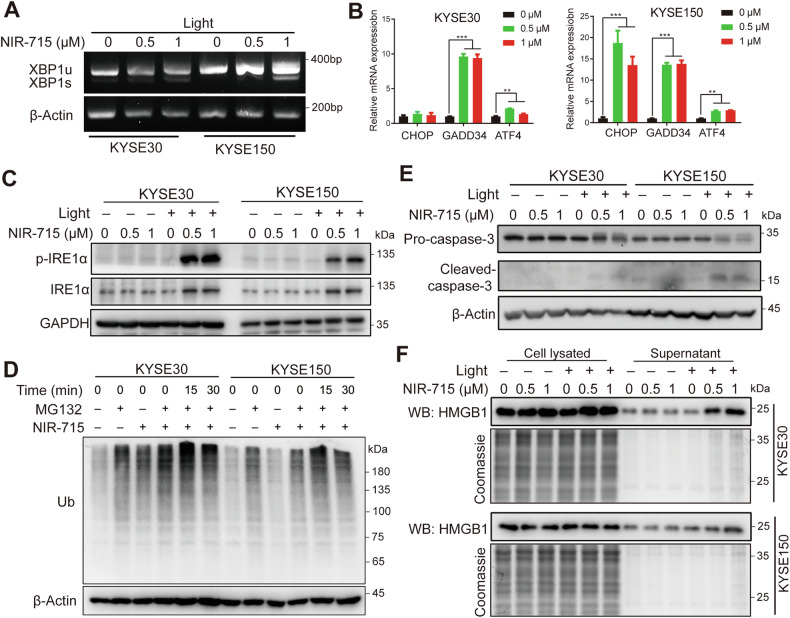
ER stress and immunogenic cell death (ICD). **A** KYSE30 and KYSE150 cell XBP1 alternatively spliced isoform (XBP1u and XBP1s) expression, after exposure to different concentrations of NIR-715 2 h and visible light exposure 10 min, cells were cultured for another 60 min before RNA extraction and analyzed by reverse-transcription PCR. **B** RT-qPCR for ER stress response genes in KYSE30 and KYSE150 cells treated with different concentrations of NIR-715 2 h and visible light exposure 10 min, cells were cultured for another 60 min before RNA extraction. **C**, **D** Lysates of KYSE30 and KYSE150 cells after treatment with NIR-715 2 h and visible light exposure 10 min, cells were cultured for another 30 min before total protein extraction and analyzed by western blotting using the indicated ER stress antibodies. **E** Western blot for cleaved caspase-3 after the same treatment with ER stress signal. **F** KYSE30 and KYSE150 cells treated with different concentrations of NIR-715 2 h and with or without visible light exposure 10 min. The the cells were cultured for another 30 min. Cell lysates and culture supernatant were collected and HMGB1 was detected by western blotting.

ER stress has been demonstrated to restore ER homeostasis and cell survival [28]. However, extreme and irreversible ER stress can lead to cell death [29]. RT-PCR analysis showed that NIR-715 induces ER stress via generation of an unconventionally spliced isoform of XBP1, denoted XBP1s, in which a 26-nucleotide fragment is excised from XBP1u mRNA, and expression of CCAAT-enhancer-binding homologous protein (CHOP), growth arrest and DNA-damage-inducible 34 (GADD34) and activating transcription factor 4 (ATF4) (Fig. 5A, B). Western blotting confirmed that phosphorylated inositol-requiring enzyme 1-α (p-IRE1α) and downstream ER-associated protein degradation were highly activated (Fig. 5C, D). These results indicate that NIR-715 induces ER stress. ICD is a cell death that can be identified by exposure of calreticulin on the surface of dying cells and release high mobility group box 1 (HMGB1) into the extracellular environment [11]. Several studies have demonstrated the relationship between ER stress and ICD [13, 16]. Treatment of cells with NIR-715 followed by visible light exposure caused cleavage of caspase-3 and HMGB1 release (Fig. 5E, F). We further used ER activators in combination with NIR-715 and found that ER activator Thapsigargine can synergize with NIR-715 to promote cytotoxicity (Supplementary Fig. S8A). However, the ER stress inhibitor 4-Phenylbutyric acid (4-PBA) was not effective in blocking the cells death although it can diminish the phosphorylation of IRE1α (Supplementary Fig. S8B, C). 4-PBA inhibits cell growth in a concentration-dependent manner, potentially by suppressing the function of histone acetylase HDAC [30] (Supplementary Fig. S8D). Finally, we used ROS inhibitor N-Acetylcysteine (NAC) combined with NIR-715, which results in reduced cell death in KYSE30 and KYSE150 (Supplementary Fig. S8E). These results indicating that NIR-715 triggers PDT can induce severe ER stress and ER-associated ICD.

### NIR-715 induces an antitumor immune response

**Fig. 6:**
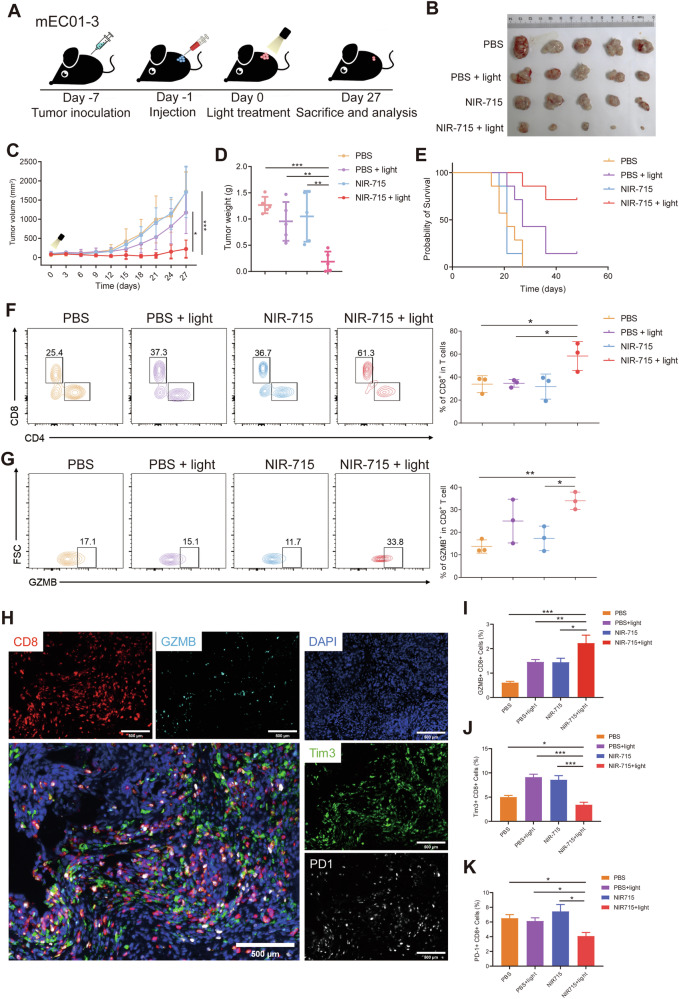
NIR-715-induced immune responses in mice bearing mEC01-3 tumors. **A** Schematic illustration of the mEC01-3 tumor model experimental design. **B** The tumor images obtained from tumor-bearing C57BL/6 mice on day 27 after NIR-715 with or without light treatment. **C**, **D** After establishment of subcutaneous mEC01-3 syngeneic mouse tumor model, tumor volumes were measured (**C**), along with tumor weights (**D**). Data are shown as mean ± SEM, *n* = 5, significance was determined by two-way ANOVA with Bonferroni’s multiple comparisons test. For tumor weight, significance was determined by unpaired two-tail Student’s t-tests. **E** Survival of mEC01-3-bearing mice (*n* = 7 independent samples). **F**, **G** Proportions of CD8^+^ T cells and GZMB^+^ CD8^+^ T cells in the TME were quantified by flow cytometry. Data represent mean ± SEM. **H** Representative multiplex immunofluorescent staining image showing distribution of CD8, PD-1, Tim-3 and GZMB expression cells in mEC01-3 syngeneic mouse tumor model. Scale bar: 500 μm. **I**–**K** Proportions of GZMB^+^ CD8^+^ T cells, Tim3^+^ CD8^+^ T cells and PD-1^+^ CD8^+^ T cells in mEC01-3 syngeneic mouse tumor model were examined on day 27 after NIR-715 treatment. Data represent mean ± SEM (*n* = 5). *P < 0.05, **P < 0.01, ***P < 0.001.

To examine antitumor immune microenvironment remodeling induced by NIR-715, we used mEC01-3 cells (Supplementary Fig. S9) to generate C57BL/6 tumor-bearing mice to further validate whether PDT can increase TILs. When the tumor volumes reached 100 mm^3^, mEC01-3-tumor-bearing mice were randomly four grouped and each mouse was treated with an intratumor injection of PBS or NIR-715, followed by visible light exposure the next day (Fig. 6A). Combination of NIR-715 with visible light exposure showed inhibition of increases in tumor volume and tumor weight, and led to longer-term survival (Fig. 6B–E). To characterize antitumor immune response activation induced by NIR-715 PDT, we analyzed the response of immune cells in tumors after mice had been treated for 27 days. First, we identified the individual population of CD4^+^ T cells, CD8^+^ T cells and GZMB^+^ CD8^+^ T cells, in the tumors, using a flow cytometry. T lymphocytes, especially the GZMB^+^ CD8^+^ CTLs are crucial in the immune response of antitumor therapy [31]. Populations of CD8^+^ T cells and GZMB^+^ CD8^+^ T cells in the NIR-715+light group were up-regulated when compared to the other three groups (Fig. 6F, G), indicative of NIR-715 PDT-mediated enhancement of cytotoxic T cell infiltration into mEC01-3 tumors. We also analyzed the population of GZMB^+^ CD8^+^ CTLs, PD-1^+^ CD8^+^ T cell and Tim3^+^ CD8^+^ T cell infiltration using multiplexed immunofluorescence staining to characterize the abundance of exhausted CD8^+^ T cells. The number of PD-1^+^ CD8^+^ T cells and Tim3^+^ CD8^+^ T cells was lower in NIR-715+light tumors compared with other three groups, and the number of GZMB^+^ CD8^+^ T cells was consistent with the flow cytometry results (Fig. 6H–K). Multiplexed immunofluorescence staining of other immune cell infiltration revealed no significant differences in regulatory T cells and dendritic cells, while macrophage showed an obviously increase in NIR-715 + light group compared other groups (Supplementary Fig. 10). The above results showed potent anticancer efficacy of the NIR-715 PDT. In addition, the body weights and H&E images of the main organs (heart, liver, spleen, lung, and kidney) from each of the four groups showed negligible differences (Supplementary Fig. 11A, B), suggesting the safety of NIR-715.

### In vivo validation of NIR-715 PDT efficacy in B16F10 tumors

**Fig. 7:**
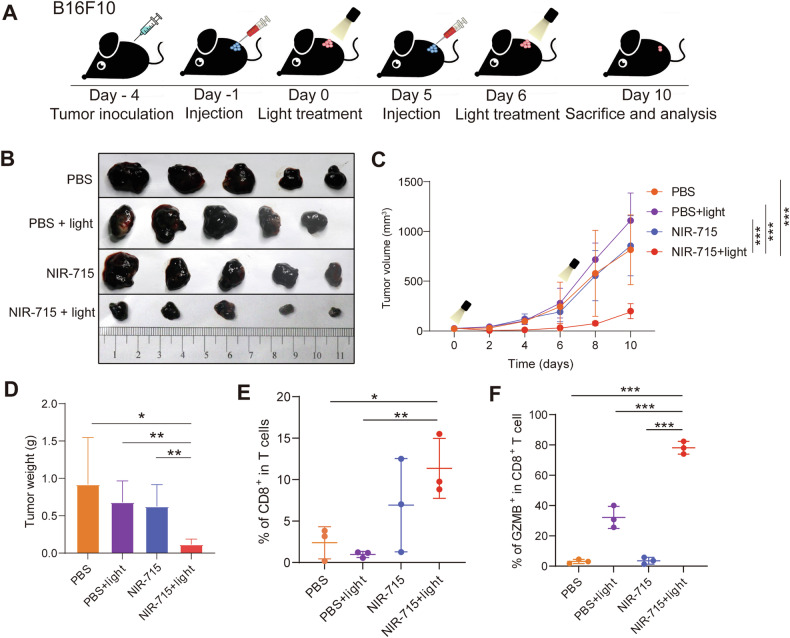
In vivo validation of NIR-715 PDT efficacy in B16F10 tumors. **A** Schematic illustration of the B16F10 tumor model experimental design. **B** Images of B16F10 tumors from tumor-bearing C57BL/6 mice on day 10 after performing NIR-715 PDT twice. **C** Tumor growth expressed as mean tumor volumes. Data are shown as mean ± SEM, *n* = 5. **D** Tumor weights of the four treatment groups. **E**, **F** Proportions of CD8^+^ T cells (**E**) and GZMB^+^ CD8^+^ T cells (**F**) in B16F10-bearing mice tumors were quantified by flow cytometry. Data represent mean ± SEM. *P < 0.05, **P < 0.01, ***P < 0.001.

We determined whether the NIR-715 induced antitumor response is broadly applicable in treating “immunologically cold” tumors, such as B16F10, a melanoma tumor that displays scarce T-cell infiltration. Similar to the mEC01-3 model, 4 × 10^5^ B16F10 cells were implanted subcutaneously into the right flank of male C57BL/6 mice and tumor volume was monitored every 2 days. The tumor growth of the NIR-715 + light group was inhibited by visible light exposure on days 0 and 6 post injection of NIR-715 (Fig. 7A, B). In addition, the body weights and main organs (heart, liver, spleen, lung kidney) from all groups showed negligible differences (Supplementary Fig. 12A, B). As shown in Fig. 7C, D, the NIR-715 or light single treatment groups showed little tumor inhibition. Thus, the NIR-715+light group showing an antitumor effect suggests that sufficient ROS production is important. To investigate activation of antitumor immunity after NIR-715 PDT, the populations of CD8^+^ T cells and GZMB^+^ CD8^+^ T cells were quantified using flow cytometry on day 10. The NIR-715 + light group showed the highest population of CD8^+^ T cells and GZMB^+^ CD8^+^ T cells in tumor tissue (Fig. 7E, F and Supplementary Fig. 12C). These results suggest that NIR-715 PDT can sensitize immune “cold” tumors by recruiting TILs in the TME.

### Inhibition of the distal tumors by NIR-715 PDT

**Fig. 8:**
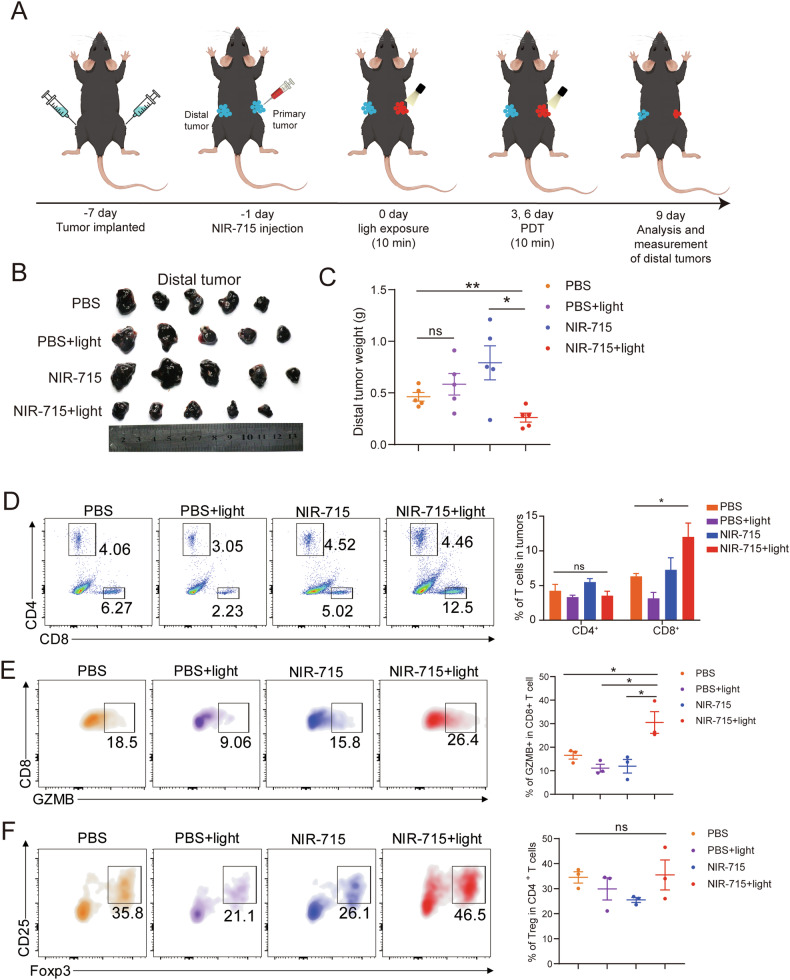
Inhibition of the distal tumors growth by NIR-715 PDT. **A** Schematic illustration of the C57BL/6 experimental design for distal tumors. The B16F10 cells were implanted on both sides, 4 × 10^5^ on the right and 2 × 10^5^ on the left flank. After 6 days, right flank tumors were injected with PBS (1% DMSO), PBS (1% DMSO) plus visible light exposure, NIR-715 (12.5 μg/kg) or NIR-715 (12.5 μg/kg) plus light directly into the tumor area and three treatment trials 9 days after injection. Left flank tumor was used for follow-up analysis. **B** The distal tumors (left flank) images obtained from the tumor-bearing C57BL/6 mice on day 9 after treatment. **C** The distal tumor weight obtained from the tumor-bearing C57BL/6 mice on day 9 after treatment. Date represent mean ± SEM (*n* = 5 biologically independent samples). **D** Representative flow cytometry plots showing four groups of CD4^+^ and CD8^+^ T (gated on CD45^+^ T cells) (left). Date represent mean ± SEM (*n* = 3 biologically independent samples). **E** Representative flow cytometry plots showing four groups of GZMB + CD8 + T (gated on CD8^+^ T cells) (left). Date represent mean ± SEM (*n* = 3 biologically independent samples). **F** Representative flow cytometry plots showing four groups of FOXP3^+^ and CD25^+^ double positive T cells (gated on CD4^+^ T cells) (left). Date represent mean ± SEM (*n* = 3 biologically independent samples). *ns*. represent *P* > 0.05, *P < 0.05, **P < 0.01.

We have also examined whether the activation of anti-tumor immune microenvironment by NIR-715 PDT were strong enough to inhibit inaccessible tumors. In this experiment, we implant two tumors on either flank and only treat right flank (primary tumors) with NIR-715 and light, then left flank (distal tumors) size and immune infiltration were measured (Fig. 8A). After primary tumors treatment, the weight change of the distal tumors was recorded and plotted (Fig. 8B, C). Surprisingly, the weight of distal tumors was decreased on the mice treated with NIR-715 + light. However, the effect of single treatment, i.e., light or NIR-715, was no effect on the distal tumors, suggesting that systemic immunity was likely restricted of the distal tumors. To better uncover the tumor inhibition effect of NIR-715 PDT strategy on distal tumors, the infiltration of GZMB^+^CD8^+^ T cells and Tregs were examined by flow cytometry. The frequency of total CD8 + T cells in NIR-715 + light group achieved increased significantly (2-fold) than those of the single treatment group, but the frequency of CD4 + T cells was no significant (Fig. 8D and Supplementary Fig. S13). Meanwhile, the frequency of GZMB^+^ T cells in the total CD8^+^ T cells was much higher in NIR-715 + light group than the other three groups (Fig. 8E). There is no difference in Treg in total CD4 + T cells (Fig. 8F). In addition, the glutamic-pyruvic transaminase (GPT), glutamic oxalacetic transaminase (GOT) and creatinine (Cr) in the mice blood from all groups showed negligible differences (Supplementary Fig. 14). These results verify that the NIR-715 PDT significantly reversed the immune tolerance of the distal tumors and enhanced antitumor immune responses.

## Discussion

Recent developments in cancer diagnosis and therapeutic strategies, including nuclear magnetic resonance imaging (NMR) [32, 33], computed tomography (CT) [34] and fluorescent probe imaging [35], real-time tumor monitoring and therapeutic effect evaluation, has allowed individualized treatment and greatly improved the efficacy of cancer treatment while reducing therapeutic side effects [36]. Fluorescence bioimaging in the near-infrared, within the wavelength range of 700–900 nm, has garnered much attention due to its deep tissue penetration, reduced photon scattering, and high resolution [37]. However, there are limited NIR probes that can be used for both tumor imaging and PDT. Here, we have designed a NIR-715 probe that shows prominent uptake and retention in tumor cells, enabling tumor NIR imaging and PDT (Fig. 2A). The pyridinium moiety in NIR-715 does not provide strong electron withdrawing effect and thus push the emission to NIR region by constructing a typical D-π-A structure, but also promised the probe good solubility and, more importantly, positive charge in the skeleton. Consequently, it was supposed that the NIR-715 was continuously attracted by the negatived charged membranes cell membranes and membranes of organelle like mitochondria and endoplasmic reticulum. Comparing to the weak emission in water and buffer solutions, the hydrophobic environment of the membrane significantly enhanced emission of NIR-715. As a result, the stronger and stronger emissions from the cell were observed after addition of the probe NIR-715 (Supplementary Fig. S4). NIR-715 induces ROS generation and promotes tumor ER stress and ICD to inhibit tumor growth.

Cold tumors are considered to be immune tolerant due to immunosuppression within the TME, and several strategies have been developed to convert a cold tumor immune microenvironment into a hot one [38]. In cold tumors, tumor cells within the TME always express diverse immunosuppressive ligands, such as PD-L1 and CTLA-4, to inhibit antitumor immunity, resulting in a lack of immunogenicity and immune cell infiltration [31]. Recent research has begun to uncover potential strategies to overcome these challenges. One approach involves combining immunotherapy with other treatment modalities, such as radiation, chemotherapy, or PDT, to enhance the immune response against the tumor [39]. In our results, compared with PBS or individual treatment, the amount of activated CD8^+^ T cells increased and exhausted CD8^+^ T cells decreased following NIR-715 plus light treatment (Figs. 6H, 7E, F), suggesting a robust immune response. The distinct change in cell type and number of infiltrating T cells can effectively enhance the anti-tumor immune response.

Several tumor immunotherapies have leveraged ICD to enhance their therapeutic effect [40]. These include agents that trigger apoptosis, necroptosis or pyroptosis, all of which can lead to ICD. ICD is a specialized form of cell death that not only eliminates tumor cells, but also alerts the immune system to the presence of a threat [40]. This process releases damage-associated molecular patterns (DAMPs) and cytokines that stimulate an immune response. In our study, NIR-715 induced cell membrane blebbing and bursting, increasing DAMP release (Fig. 4E, F). HMGB1 release within the TME enhances a signal that favors the maturation of DCs [11]. Recognition of DAMPs by antigen-presenting cells, such as DCs, further activates the adaptive immune system, leading to the generation of tumor-specific T-cells.

However, the induction of ICD is not always successful or sufficient to eliminate the tumor. The immune response triggered by ICD can be dampened by various immunosuppressive mechanisms within the TME. This highlights the need for combinatorial approaches that simultaneously target multiple immunosuppressive mechanisms and enhance ICD induction [11]. The ER is a site of protein processing, and modification in eukaryotic cells, and plays an important role in maintaining protein homeostasis [41]. ROS generation mediated by NIR-715 could cause severe ER stress, as shown by upregulation of the proapoptotic protein CHOP, GADD34 and ATF4. Moreover, dramatic ER stress induces an unfolded protein response and consequent exposure of calreticulin on the cell surface, as well as cell death and HMGB1 release [11]. Activation of NIR-715 disrupted ER structure and induces ER stress, which triggered bona fide ICD in cancer cells (Fig. 4D and Fig. 5A–C). In mEC01-3 syngeneic models, light exposure alone group, the tumor weight of some mice decreased but there is no significant difference compared with the PBS group (*P* = 0.121). Considering the reduction in tumor weight in PBS group, we think there are two possibilities. On the one hand, there are differences between individual mice, and on the other hand, ten minutes of light exposure will generate heat in the tumor, there is photothermal therapy. In the current study, the mild photothermal therapy is important in sensitizing the immunodeficient tumors as well as potentiating the anti-PD-L1 treatment [42]. In KYSE30 xenograft, because the BALB/c nude mice are immune deficient, slight light exposure has no effect. Moreover, NIR-715 exhibits low long-term toxicity, and our assessments through mouse weight and histological HE staining of mouse tissues (Supplementary Figs. S11, S12) and blood biochemical index GPT, GOT, Cr have shown no significant toxicity (Supplementary Fig. S14). This data supports the further development and potential clinical application of NIR-715 as a novel PDT agent.

Activation of anti-tumor immunity by immunogenic cell death not only can kill residual tumor cells after treatment, but also prevent tumor recurrence and metastasis [43, 44]. In addition, immunogenic cell death can also enhance the sensitivity of tumor cells to other treatments, such as radiotherapy, chemotherapy, and immunotherapy. Esophageal cancer was one of the first clinical indications for PDT to be approved as an endoscopic procedure in the United States and Japan [45]. H. Sato et al. showed that PDT combined with endoscopic resection improves the prognosis of local recurrence in ESCC patients [46]. Recent studies have highlighted the synergistic potential of combining PDT with immunotherapy. PDT can induce inflammation at the treatment site, leading to the activation of myeloid cells and a significant effect on the immune system [47]. This can result in the release of tumor antigens, which can stimulate an acute inflammatory response and activate lymphoid cells, leading to tumor-specific immunity [48]. However, a significant limitation of NIR-715 is the limited penetration of light into deep-seated tumors. Our study is limited to treating subcutaneous tumors that are superficial, and mild skin lesions occurred in our mice after treatment. But NIR-715 has limitations in its application range due to its requirement for intratumoral injection. X Wang, et al. developed a kind of photoactivatable probe, THTTPy-PTSA, which enables targeting of the ER and mitochondria and enhanced the antitumor immune efficacy [49]. The ability of NIR-715 to stimulate an antitumor immune response could be particularly useful in a clinical setting where the goal is to target both the primary tumor and metastatic lesions. Our results provide evidence of the potential value of NIR-715 in remodeling the tumor immune microenvironment and tumor combination therapy.

In conclusion, we successfully developed a near-infrared probe with a prominent uptake and retention capacity in tumor cells for cancer NIR imaging and PDT. Upon being activated by light, NIR-715 specifically induces ROS generation and accumulates near the ER to promote tumor ER stress and ICD, resulting in tumor inhibition. Because of the induction of ICD in tumor tissues, this probe could robustly induce an anti-tumor immune response by enhancing the number of CD8^+^ tumor-infiltrating T lymphocytes in the TME, showing good immune antitumor efficacy for tumor inhibition.

## Materials and Methods

### Reagents

SYTOX™ Green Dead Cell Stain (S34860) was purchased from Thermo Fisher. 4-Nitroquinoline-1-oxide (4-NQO, N8141) was from Sigma. 4-Phenylbutyric acid (HY-A0281), Thapsigargin (HY-13433), Acetylcysteine (HY-B0215) were purchase from MedChemExpress. Primary antibodies were anti-CD8α (98941), anti-CD11c (97585), anti-F4/80 (70076), anti-Foxp3 (12653), anti-CD4 (25229), anti-GZMB (44153) from Cell Signaling Technology. anti-PD-1 (ab214421, Abcam) and anti-Tim-3 (ab241332, Abcam). A fluorescent ER localization reporter plasmid, denoted pBobi-GFP-ER, was constructed by inserting the calreticulin ER targeting sequence at the N terminus of GFP. Glutamic pyruvic transaminase (GPT) activity assay kit (AKAM006C, boxbio, Beijing, China), Glutamic oxalacetic transaminase (GOT) activity assay kit (AKAM019C, boxbio, Beijing, China), Creatinine (Cr) assay kit (D799853, Sangon Biotech, Shanghai, China)

### Cell culture

The esophageal cancer (EC) cell lines have been described previously [50]. Human EC cell lines KYSE30 and KYSE150, and 4T1 murine triple-negative breast cancer cells were grown in RPMI 1640 medium (Thermo Fisher). KYSE520, MDA-MB-453 (human breast cancer cells), MDA-MB-231 (human breast cancer cells), and B16F10 (murine melanoma) cells were grown in DMEM (Thermo Fisher) supplemented with 10% fetal bovine serum (Thermo Fisher), penicillin (100 U/mL), and streptomycin (100 g/mL), and cells were incubated at 37 °C in 5% CO_2_. The murine EC cell line, mEC01-3, was established in our laboratory (details are in Supplementary Fig. S9 and Methods). All cell lines were verified by STR analysis and to be free of mycoplasma contamination (IGEbio, Guangzhou, China).

### Synthesis of NIR-715

All chemical and reagents were purchased from Aladdin Chemical Company. Solvents were purified and dried by standard methods before use.

Synthesis of compound **1** (Fig. 1A). To a dry three-neck flask, 3.0 g of 4,7-dibromobenzo[c] [1,2,5] thiadiazole, 1.5 g of (5-formylthiophen-2-yl) boronic acid, 11.3 g of K_2_CO_3_ and 300 mL of 1,2-dimethoxyethane were combined and degassed by bubbling argon gas into the mixture for 30 min. Then, 180 mg of tetrakis (triphenylphosphine)palladium was added to serve as a catalyst. After bubbling argon gas for an additional 20 min, the reaction was moved to an oil bath and stirred at 80 ^o^C for 16 h. The reaction solution was concentrated on a rotatory evaporator, followed by extraction with CHCl_3_/H_2_O. The organic layer was collected and dried on anhydrous MgSO_4_. After concentration, the solution was separated by chromatography, using petroleum ether and ethyl acetate as solvent, to give a yellow oily solid (0.6 g). ^1^H NMR (400 MHz, CHCl_3_-d) δ 9.99 (*s*, 1H), 8.19 (*d*, *J* = 3.0 Hz, 1H), 7.92 (*d*, *J* = 7.7 Hz, 1H), 7.89–7.82 (*m*, 2H).

Synthesis of compound **2** (Fig. 1A). We combined 0.39 g of compound **1**, 0.53 g of (4-(diphenylamino) phenyl)boronic acid, 1.01 g of K_2_CO_3_ and 300 mL of 1,2-dimethoxyethane, and degassed the solution by bubbling argon gas for 30 min. After adding 0.04 g tetrakis (triphenylphosphine)palladium as a catalyst and bubbling argon gas for 20 min, the solution was transferred into an oil bath and stirred at 80 °C for 16 h. The reaction solution was concentrated on a rotatory evaporator and was extracted by using CHCl_3_/H_2_O. The organic layer was collected and dried on anhydrous MgSO_4_. After concentration, the solution was separated on chromatography by using petroleum ether and ethyl acetate as solvent to yield a red solid (0.40 g). ^1^H NMR (400 MHz, CHCl_3_-d): δ 9.98 (*s*, 1 H), 8.23(*d*, *J* = 4.0 Hz, 1 H), 8.07 (*d*, *J* = 7.6 Hz, 1 H), 7.88-7.91 (*m*, 2 H), 7.86 (*d*, *J* = 4.0 Hz, 1H), 7.76 (*d*, *J* = 4.0 Hz, 1H) 7.28-7.73 (*m*, 4 H), 7.18–7.22 (*m*, 6 H), 7.07-7.11 (*m*, 2 H).

Synthesis of compound **3** (Fig. 1A). We added 5.0 g of 4-picoline and 6.67 g of 2-bromoethanol into 100 mL of acetonitrile and stirred at 80 °C for 16 h. After the reaction, the solvent was removed by vacuum to give a pure product of 3 (11.0 g). ^1^H NMR (400 MHz, DMSO-*d6*) δ 8.93-8.98 (*m*, 2H), 8.02-8.05 (*d*, 2H), 5.26 (*s*, 1H), 4.66-4.71 (*t*, 2H), 3.85-3.87 (*m*, 2H), 2.64 (*s*, 3H).

Synthesis of compound NIR-715 (Fig. 1A). For NIR-715 synthesis, 0.20 g of compound **2** and 0.88 g of compound **3** were added into 50 mL ethanol, followed by addition of 3 drops of piperidine as catalyst. The reaction mixture was stirred at 80 ^o^C for 16 h. After the reaction, the solution was concentrated and subjected to a silica column by using dichloromethane and ethanol as eluent. A black solid product was obtained (0.15 g). ^1^H NMR (400 MHz, DMSO-d6) δ 8.87 (d, J = 6.4 Hz, 2H), 8.35-8.24 (m, 4H), 8.21 (d, J = 4.0 Hz, 1H), 8.00 (d, J = 8.7 Hz, 2H), 7.94 (d, J = 7.6 Hz, 1H), 7.66 (d, J = 4.0 Hz, 1H), 7.40-7.32 (m, 5H), 7.15-7.08 (m, 8H), 5.27 (m, J = 5.3 Hz, 1H), 4.56 (m, J = 4.9 Hz, 2H), 3.87 (m, J = 5.1 Hz, 2H).

#### Instrument

UV-vis spectra were recorded on an UV-4802S (UNICO, Shanghai, China) UV-Visible spectrophotometer. Fluorescence spectra were measured on a QM-TM (PTI, US) spectrophotometer. Proton and carbon nuclear magnetic resonance spectra (1H and 13 C NMR) were recorded on an AVANCE-400 MHz and 100 MHz NMR spectrometer, respectively, with TMS as an internal reference. Compounds were routinely checked by thin layer chromatography (TLC) on silica gel plates using petroleum ether (PE)/ethyl acetate (EA) or chloroform (DCM)/methanol (MeOH). The crude products were purified by flash column chromatography and re-crystallization techniques. Quantum computations were conducted by using the DFT method at B3LYP/6-311 G (d, p) and B3LYP/CAM-6-311 + G (d, p) levels on Gaussian software. A common white light was employed as the excitation light source for imaging and photodynamic treatment.

### RNA extraction and quantitative real-time PCR (qRT-PCR)

RNA extraction and qPCR were performed as previously described [51]. RNA was reversed transcribed and quantitative PCR was performed using a StarScript II RT kit (A214, GenStar) and 2×RealStar Green Fast Mixture with ROX II (A304, GenStar). Primer sequences used for quantitative PCR are listed in Supplementary Table 1. ACTB was used as an internal reference and for normalization. The CT value was normalized using the formula: ΔCT = CT (target gene) – CT (ACTB). Relative mRNA expression was normalized against the relative value obtained from the control group using the formula: ΔΔCT = ΔCT (treatment group) – ΔCT (control group). The expression FC was determined according to the following formula: FC = 2 − ΔΔCT. All experiments were performed in triplicate.

### Western blotting

Total cell lysates were extracted using 1× SDS sample buffer (50 mM Tris-HCl, 2% SDS, 0.1% bromophenol blue, 10% glycerol, 1% β-mercaptoethanol). Experimental procedures were performed according to standard methods [52]. Primary antibodies included anti-pIRE1α (Ser724) (NB100-2323SS, Novus Biologicals), anti-IRE1α (NB100-2324SS, Novus Biologicals), anti-pro-caspase-3 (14220, Cell Signaling Technology), anti-cleaved-caspase-3 (9664, Cell Signaling Technology), anti-HMGB1 (A19529, ABclonal), anti-ubiquitin (10201-2-AP, Proteintech), anti-GAPDH (60004-1-AP, Proteintech), and anti-β-Actin (66009-1-Ig, Proteintech). After incubating with HRP-conjugated AffiniPure goat anti-rabbit IgG(H + L) (SA00001-2, Proteintech) or HRP-conjugated AffiniPure goat anti-mouse IgG(H + L) (SA00001-1, Proteintech), the signals were measured with ECL reagent (sc-2048, Santa Cruz).

### Syngeneic mouse esophageal cancer cell lines established

All animal studies were conducted in accordance with protocols approved by the Animal Research Committee of the Shantou Administration Center (SUMC2022-458). Primary mouse esophageal tumors in C57BL/6 mice were established with 4-NQO according to a previous protocol [53]. In brief, six-week-old female C57BL/6 mice were fed 4-NQO (100 μg/mL in drinking water) for 16 weeks, then fed normal water for 12 weeks and examined every 3 days for weight. The mice were sacrificed, and esophagi were resected under sterile conditions. The resultant esophageal tumor tissues were washed 3 times with PBS containing penicillin (200 U/mL) and streptomycin (200 μg/mL), and cut into 1 mm^3^ pieces in DMEM containing 10% fetal bovine serum (Thermo Fisher), penicillin (100 U/mL), and streptomycin (100 μg/mL), and cells were incubated at 37 °C in 5% CO_2_ for 7 days. The cancer-associated fibroblasts were digested for 2 min in 0.25% trypsin and removed. The remaining tumor cells were washed with PBS and digested with 0.25% trypsin for 10–20 min. After the second digestion, tumor cells were resuspended in 2 mL DMEM containing 10% fetal bovine serum, and consecutively passaged for 20 generations, resulting in two established a mouse ESCC cell line, denoted mEC01-3, and verified by STR analysis.

### NIR-715 uptake assay

KYSE30 and KYSE150 were cultured in 35 mm dishes at 1 × 10^5^ cells per well, and incubated overnight. NIR-715 at 0.5, 1, and 2 μM was added and images and fluorescence intensity were obtained using a fluorescence microscope (LIONHEART^FX^, Bio Tek Instruments).

### Cell viability assay

Cells were treated with NIR-715 (0, 0.5, 1, 2, 4 μM) for 2 h. Then, growth medium was replaced with fresh culture medium and cells were treated with or without visible light exposure at 400–760 nm wavelengths (Transilluminator, Model TW-26, Upland, CA 91786 U.S.A) for 10 min. After 24 h, cell viability was determined using a CellTiter 96 AQueous One Solution Cell Proliferation Assay kit (MTS, G3581, Promega Corporation, Beijing, China) according to the manufacturer’s manual, and absorbance at 492 nm was determined using a microplate reader.

### SYTOX staining

KYSE30 cells were seeded into 12-well plates and treated with NIR-715 (2 μM) for 2 h. Then, cells were washed twice with PBS and fresh culture medium was replaced. After 10 min of visible light exposure, the cells were cultured for another 24 h. Then, 0.5 μM SYTOX was added to the medium, cells were further incubated for 10 min, and images were obtained using a Zeiss Observer A1 microscope. The results were obtained from at least three independent experiments.

### Colony formation assay

Fifty cells were seeded into each well of a 6 well plate and cultured overnight. The cells were treated with NIR-715 (0, 0.5, 1, 2 μM) for 2 h, and washed three times with PBS. Then, cells were refed with fresh culture medium and treated with or without visible light exposure for 10 min. After 7–10 days, colonies were stained with 0.5% crystal violet, and counted using ImageJ software.

### Confocal fluorescence microscopy

KYSE150 cells were seeded onto fibronectin-coated coverslips (F2006, Sigma) in 12-well plates. Cells were transfected with the GFP-ER plasmid for 24 h. Then, NIR-715 was added to the cells followed by incubation at 37 °C for 2 h. Then, medium was replaced with fresh culture medium and cells were treated with or without visible light exposure for 10 min. Sample processing was performed as previously described [54]. In brief, cells were fixed in 4% paraformaldehyde for 15 min, then washed with PBS, before 0.1% Triton X-100 was added for 10 min. Then, the cells were wash and counterstained with DAPI (D9564, Sigma-Aldrich) for 10 min. Images were obtained using a Zeiss LSM800 confocal microscope (Carl Zeiss).

### Live-cell ultra-high-resolution fluorescence microscopy

KYSE150 cells were seeded in a glass bottom petri dish (D35-20-1.5H, Cellvis). Cells were transfected with the GFP-ER plasmid for 24 h. Then, cells were incubated with NIR-715 at 37 °C for 2 h. Then, medium was replaced with fresh culture medium and cells were further cultured for 24 h. Images of ER morphology were obtained with an HIS-SIM (Guangzhou Computational Super-Resolution Biotech Co. Ltd).

### Intratumor retention of NIR-715 in nude mice

All animal studies were conducted in accordance with the protocols approved by the Animal Research Committee of the Shantou Administration Center (SUMC2022-458). For the nude mouse (Vital River Laboratories, Beijing, China) experiments, six-week-old male mice were randomly divided into two groups (five mice each) and 1 × 10^6^ KYSE30 cells were injected. In nude mice bearing tumors after 10 days following s.c. injection, 100 μL NIR-715 (25 μg/mL) was injected into each tumor. Injection of 100 μL PBS containing 1% DMSO was used for the controls. Fluorescence of NIR-715 was detected at different times (2, 5, 24, 48 h) using a Caliper IVIS Kinetic small animal in vivo imaging system (IVIS Lumina XR) with an excitation filter of 400–650 nm. At the last point in time, mice were sacrificed, and the major organs were imaged.

### Hematoxylin/eosin (HE) staining and immunohistochemistry (IHC)

HE and IHC of mice tumor tissues were performed as described previously [51]. Anti-keratin 6 A (1:400, 905701, BioLegend), anti-Ki67 (1: 200, MAB-0129, MXB Biotechnologies) and anti-cleaved-caspase-3 (Asp175) (9661, Cell Signaling Technology, 1: 400) antibodies were used. In brief, tumor tissues from nude mice were fixed in 4% paraformaldehyde for 6 h and dehydrated overnight. Fixed tissues were sectioned at 4 μm and processed for HE and IHC following standard procedures.

### Mice subcutaneous tumor formation assay

For nude mice (Vital River Laboratories, Beijing, China) experiments, six-week-old male mice were randomly divided into six groups (five mice each), and 1 × 10^6^ KYSE30 were injected subcutaneously (s.c.) into the right flank. Body weight and tumor size were examined every 3 days. Three days after subcutaneous transplantation, PBS (1% DMSO) or NIR-715 (12.5 μg/kg and 25 μg/kg) were injected into the tumor. After 24 h, tumors were exposure with or without visible light at 400–760 nm wavelengths (1.0 W/cm^2^, BaiNa lighting appliance factory, Shenzhen, China) for 10 min, after which tumor size was measured every 3 days, and volume was calculated as (length×width^2^)/2. On day 14, all mice were sacrificed, and tumors were weighed. In addition, HE and IHC were carried out to characterize tumor cell proliferation and apoptosis.

#### NIR-715 efficacy on murine esophageal tumor models and B16F10 melanoma tumors

To evaluate the potency of NIR-715 for cancer therapy, a syngeneic tumor models were performed using C57BL/6 mice injected with mEC01-3 EC cells or B16F10 cells. For these experiments, 4 × 10^6^ mEC01-3 cells or 4 × 10^5^ B16F10 cells were injected s.c. into the right flanks of C57BL/6 mice. After tumors reached 100 mm^3^, mice in groups 1-4 were intratumor injected with PBS (1% DMSO), PBS (1% DMSO) plus visible light exposure, NIR-715 (12.5 μg/kg) or NIR-715 (12.5 μg/kg) plus light directly into the tumor area and two treatment trials (B16F10 tumor model) six days after injection. Tumor size and body weight were monitored every 3 days (mEC01-3 model) or 2 days (B16F10 model). For distal tumors inhibition experiment, according to previous study [42], 4 × 10^5^ B16F10 cells were injected s.c. into the right flanks and 2 × 10^5^ B16F10 cells were injected s.c. into the left flanks of C57BL/6 mice. After right flank tumors reached 100 mm^3^, mice in groups 1-4 were intratumor injected with PBS (1% DMSO), PBS (1% DMSO) plus visible light exposure, NIR-715 (12.5 μg/kg) or NIR-715 (12.5 μg/kg) plus light directly into the right flank tumor area and three treatment trials 9 days after injection. Left flank tumor was used for follow-up analysis. Tumor volume was calculated using the following formula: (length×width^2^)/2. After PDT, representative mice were sacrificed and the tumor, blood, and main organs (including heart, liver, kidney, lung, and spleen) were collected for follow-up experiments. Finally, the survival time of the remaining mice was determined, with mice being euthanized when tumor size exceeded 800 mm^3^, loss of 20% body weight or when the experiment was terminated on day 45.

### Flow cytometry analysis

Cellular ROS was measured using a Reactive Oxygen Species Assay Kit (50101ES01, Yeasen, Shanghai, China). KYSE30 and KYSE150 cells were cultured in 6-well plates and treated with NIR-715 as indicated. After 2 h, the cells were refed with fresh culture medium and exposure with visible light for 10 min. Then, cells were incubated with DCFH-DA (2, 7–dichlorodihydrofluorescein diacetate) for 10 min at 37 °C. ROS generation was analyzed using a BD Accuri C6 flow cytometer. For the TME immune cell infiltration after NIR-715 PDT, GZMB^+^ CD8^+^ T cells and CD8^+^ T cells were analyzed by flow cytometry (CYTEK Aurora). To evaluate cytotoxic T lymphocyte activity after NIR-715 treatment, tumor tissues were minced into 1 mm^3^ pieces and dissociated with 200 U/mL collagenase (17100017, Thermo Fisher) for less than 1 h at 37 °C, then dissociated tissue was shaken at 200 rpm. After incubation, DMEM (10% FBS) was added, and suspended cells were passed through a 40 μm cell strainer and recovered by centrifugation at 350 × *g* for 8 min. Cells were resuspended and stained with Fixable Viability Stain 700, APC-Cy7-conjugated anti- mouse CD45 (30-F11, BD Pharmingen), BV510-conjugated anti-mouse CD3 (145-2c11, BD Pharmingen), PerCP-Cy5.5-conjugated anti- mouse CD8 (53-6.7, BD Pharmingen), PE-conjugated anti- mouse granzyme B (NGZB, Thermo Fisher), and FITC-conjugated anti- mouse CD4 (RM4-5, BD Pharmingen), PE-eFluor® 610-conjugated anti-mouse Foxp3 (61-5773-82, Thermo Fisher), APC-conjugated anti-mouse CD25 (557192, BD Pharmingen) according to the manufacturer’s instructions. All antibodies used in our experiments were diluted 100-fold.

### Multiplex immunofluorescence staining and cell-type quantification

Multiplex immunofluorescence staining and analysis were performed as described previously [55]. Tumors were fixed in 4% paraformaldehyde for 12 h at 4 °C and dehydrated overnight. Fixed tissues were sectioned at 4 μm thickness, dewaxed in xylene, rehydrated in 100% ethanol for 5 min, 90% ethanol for 5 min, and 70% ethanol for 5 min to distilled water for 5 min. Antigen retrieval was performed by heating in EDTA (MVS-0099, MXB Biotechnologies) for 15 min, followed by blocking peroxidase activity with 3% H_2_O_2_ solution. The sections were then blocked with 10% goat serum, and 5-plex staining was performed with a PANO 6-plex IHC kit (10081100050, Panovue, Beijing, China) according to the manufacturer’s instructions. The multiplexed assay was carried out with each section subject to four rounds of primary antibody staining, followed by horseradish peroxidase-conjugated secondary antibody incubation and tyramide signal amplification. Samples were microwave heat-treated following each round of tyramide signal amplification. After incubation with the last antibody, nuclei were counterstained with DAPI (D9542, Sigma-Aldrich) and mounted with Prolong Glass Antifade Mountant (P36981, Thermo Fisher).

Multispectral images were obtained using the Polaris System (PerkinElmer, Massachusetts, USA). Positively-labeled PD-1^+^ CD8^+^ T cells, Tim3^+^ CD8^+^ T cells, and GZMB^+^ CD8^+^ T cells in the specimens were analyzed and quantified using Inform advanced image analysis software (PerkinElmer, Massachusetts, USA). We analyzed 10–15 random high-power fields (20× magnification) per sample inside the region of interest.

### Statistical analysis

Statistical analysis was performed using GraphPad Prism 9 software (GraphPad Prism Software Inc., San Diego, CA, USA). All results were acquired from three independent experiments and representative results are displayed. Statistical significance was set at *P* < 0.05 and denoted as *P < 0.05, ** *P* < 0.01, ***P < 0.001.

## Supplementary information

Supplementary Data

uncropped original western blots.

checklist

Fluorescence intensity images of KYSE150 after NIR-715 addition.

Fluorescence images of ER morphology in live cells after treatment with NIR-715.

Fluorescence images of NIR-715 in live cells.

source data

## Data Availability

The data generated in this study are available within the article and its supplementary data files. Raw data for this study were generated at Xu lab. Derived data supporting the findings of this study are available from the corresponding author upon request.
