# Supplementary material for: NIR-715 photodynamic therapy induces immunogenic cancer cell death by enhancing the endoplasmic reticulum stress response
Source: Cell Death Dis. 2024 Dec 18;15(12):890. doi: 10.1038/s41419-024-07283-4 (PMC11655639; doi:10.1038/s41419-024-07283-4)
Supplement: Supplementary file 1 — Supplementary Data [file 41419_2024_7283_MOESM1_ESM.docx]

Supporting Information for

NIR-715 photodynamic therapy induces immunogenic cancer cell death by enhancing the endoplasmic reticulum stress response

Zhen-Yuan Zheng, Wan Lin, Jia-Wan Su, Qing-Feng Huang, Cong Zhang, Wen-Xing Pan, En-Min Li, He-Feng Zhang^*^, Li-Yan Xu^*^

**
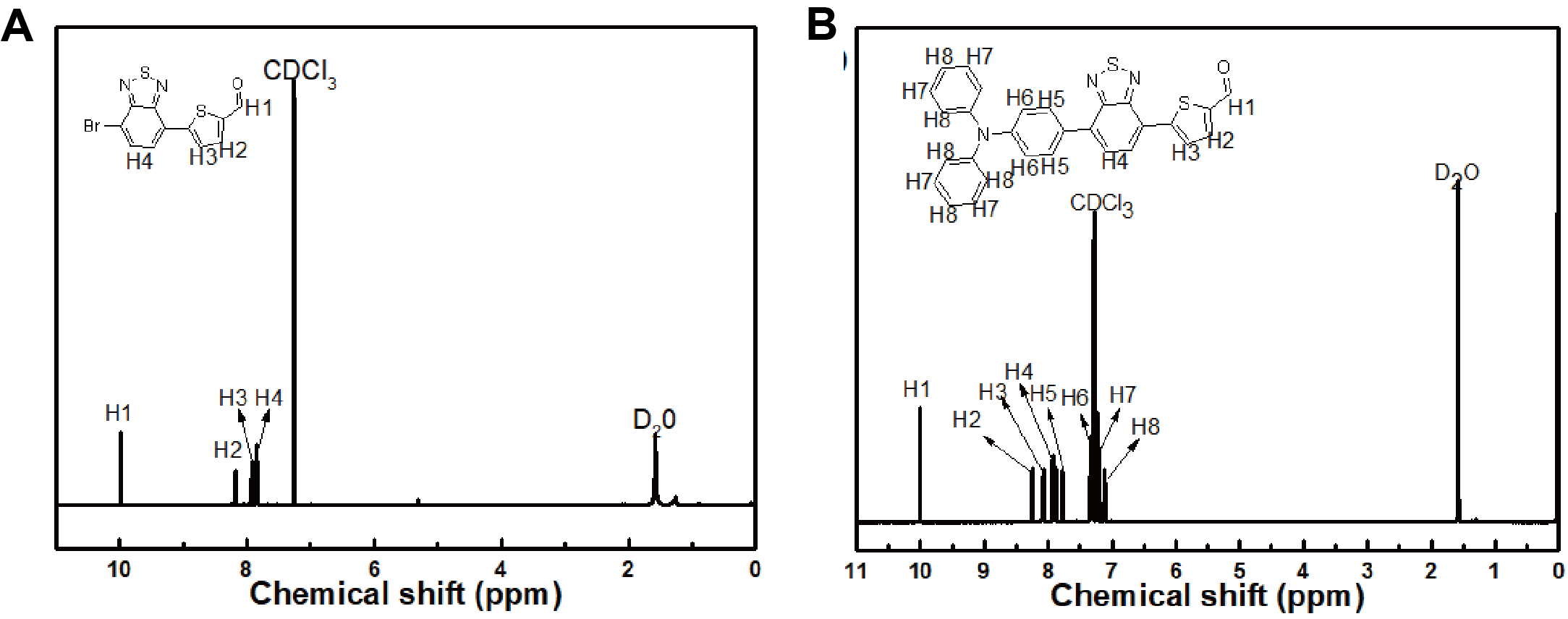
Figure S1** ^1^H NMR spectra (CDCl_3_) of a) compound1 and b) compound 2

**
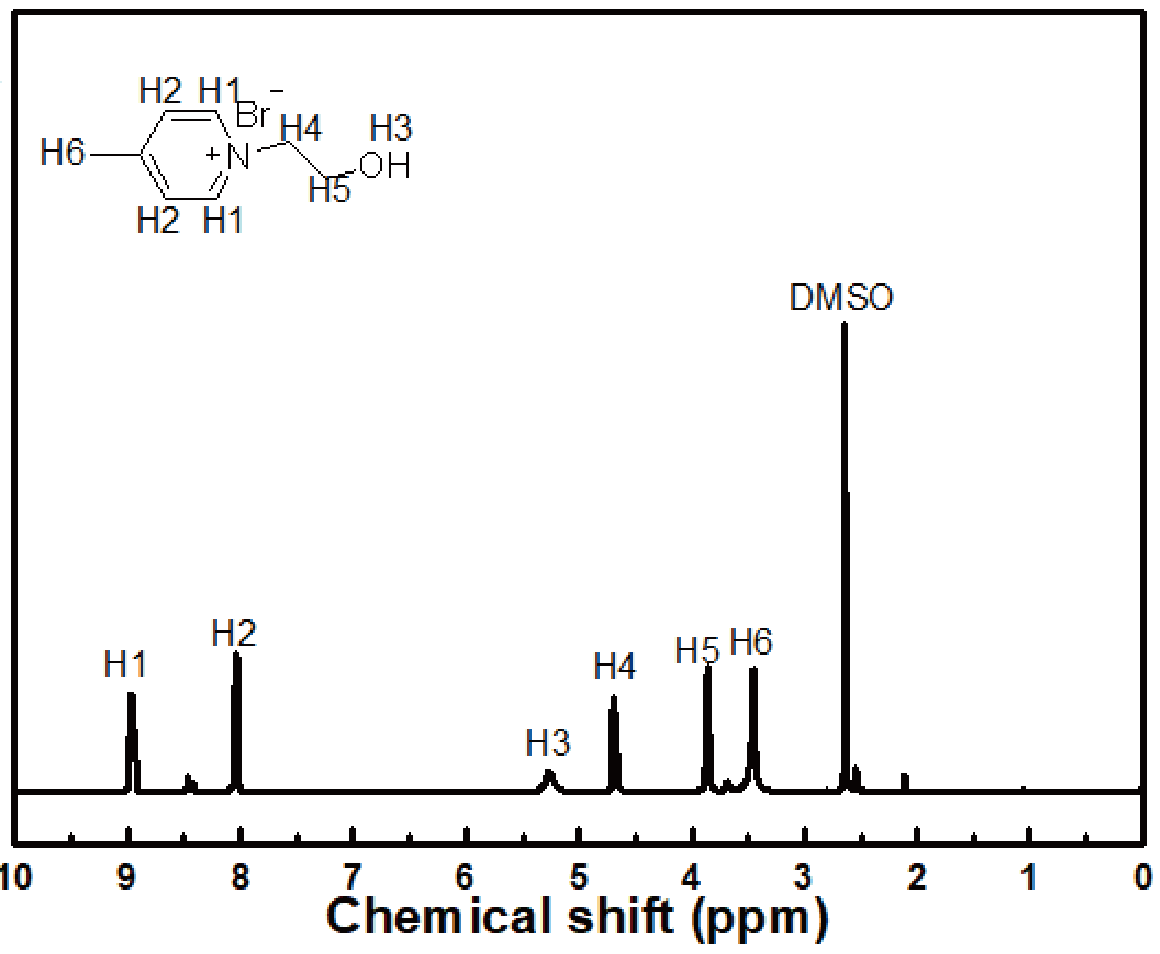
**

**Figure S2** ^1^H NMR spectrum of compound 3（DMSO-d6）

**
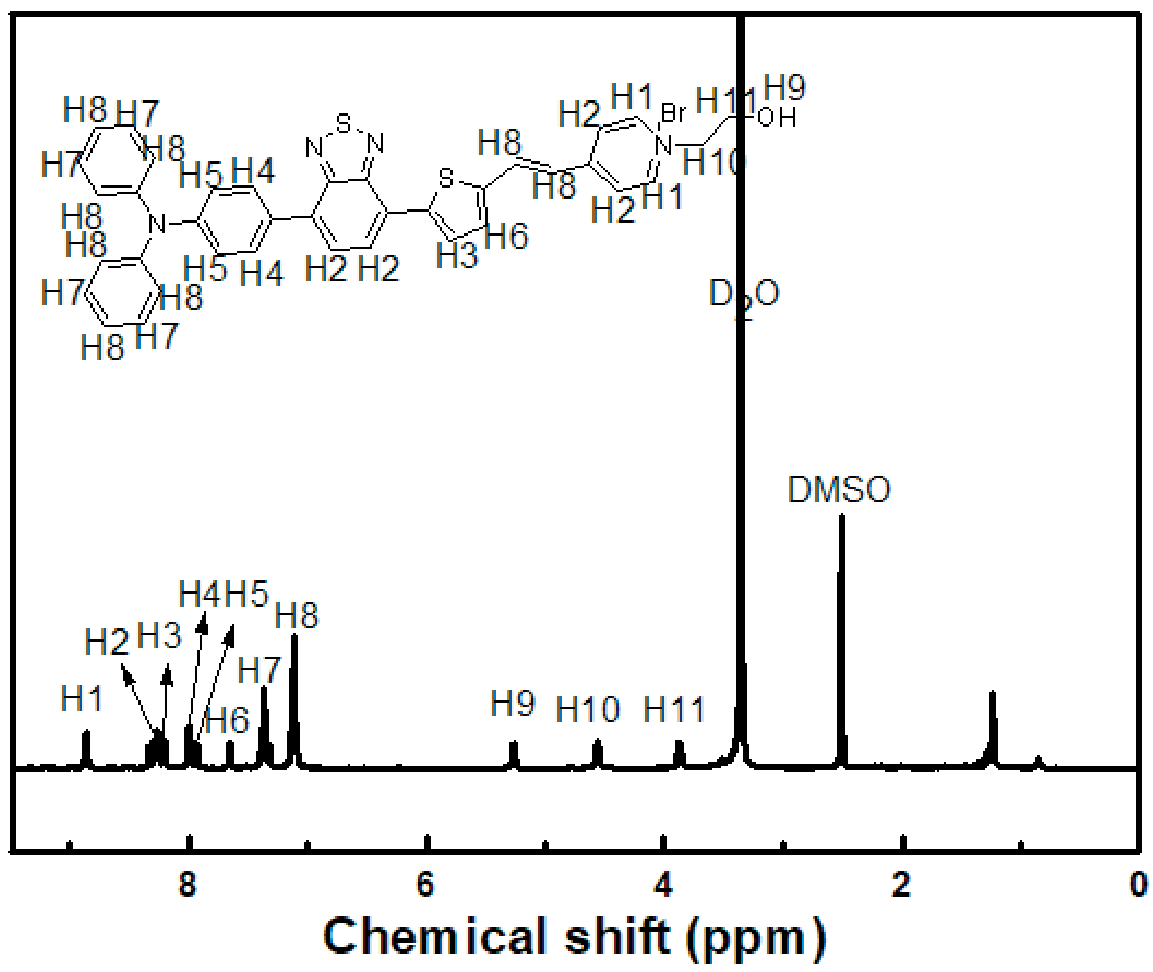
**

**Figure S3** ^1^H NMR spectrum of NIR-715（DMSO-d6.）

**
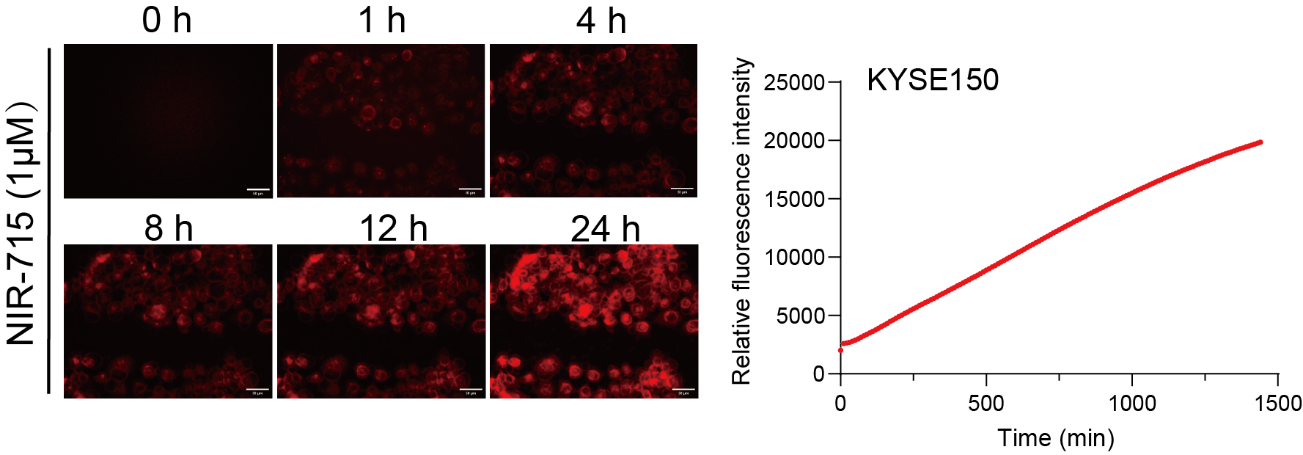
**

**Figure S4** Fluorescence intensity images of KYSE150 after 1 μM NIR-715 addition. Images were recorded every 10 min for a total of 24 hours. Scale bar: 50 μm.

**
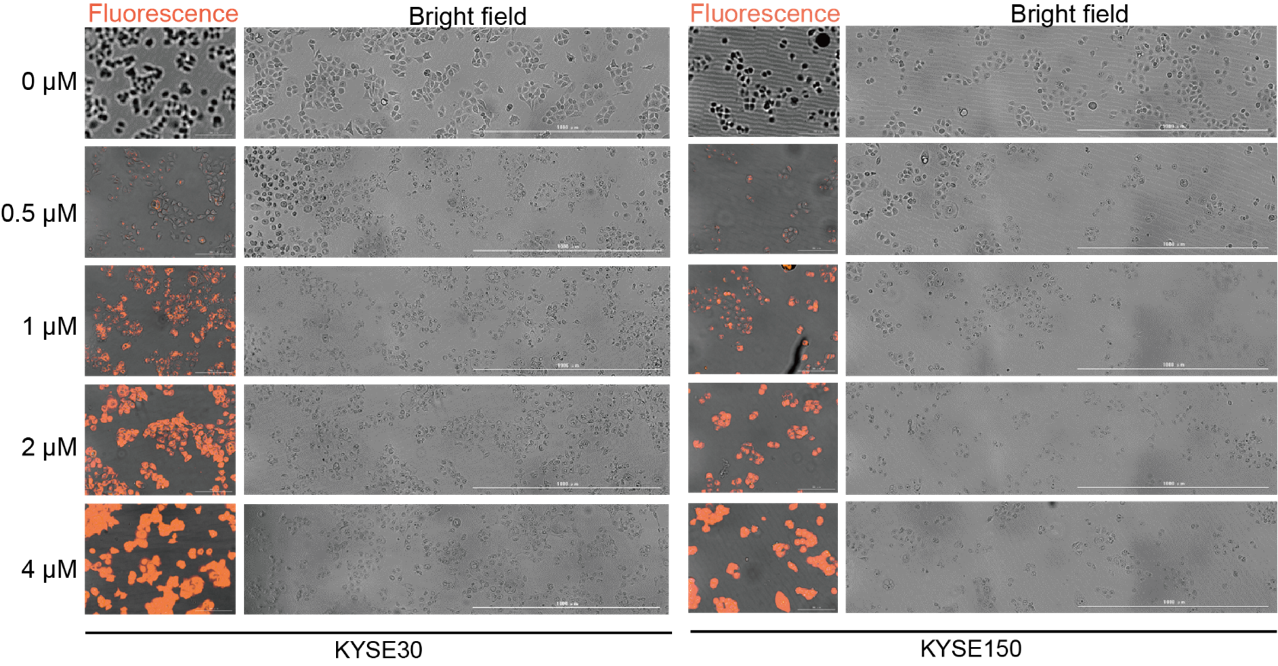
**

**Figure S5** Cell morphology was obtained under a fluorescence microscope with different concentrations of NIR-715 after 10 min light exposure and 12 h incubation. Scale bar: 1000 μm.


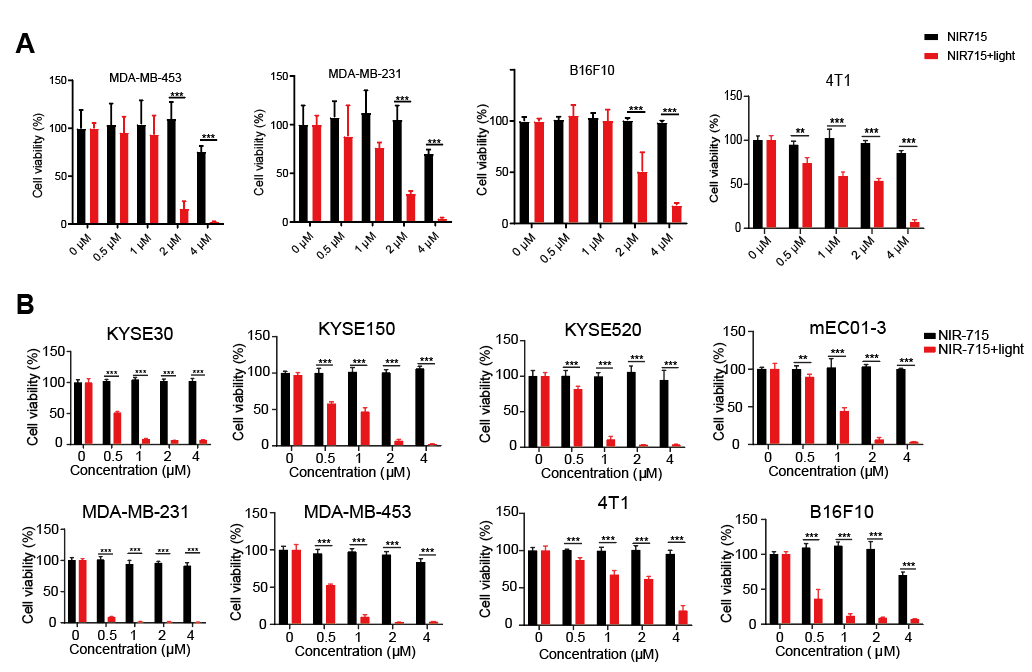


**Figure S6 (A)** Cell viability of MDA-MB453, MDA-MB-231, B16F10 and 4T1 cells treated with various concentrations of NIR-715 2 h, then cultured 12 h. The cells with or without light exposure for 10 min. Cell viability was evaluated using the MTS assay. All data are presented as the mean ± SD (n = 4). ***P* < 0.01 ****P* < 0.001.
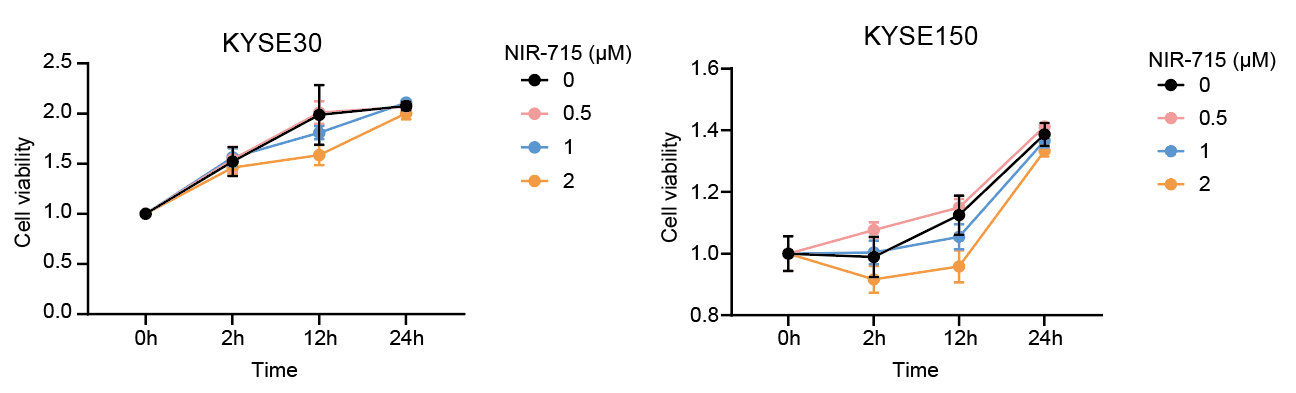


**Figure S7** KYSE30 and KYSE150 were seeded in 96 well and incubated overnight. The cells were added to the NIR-715 with indicated concentration under the conditions of no light exposure. MTS assays were performed after 0 h, 2 h, 12 h, 24 h treatment. All data are presented as the mean ± SD (n = 4).


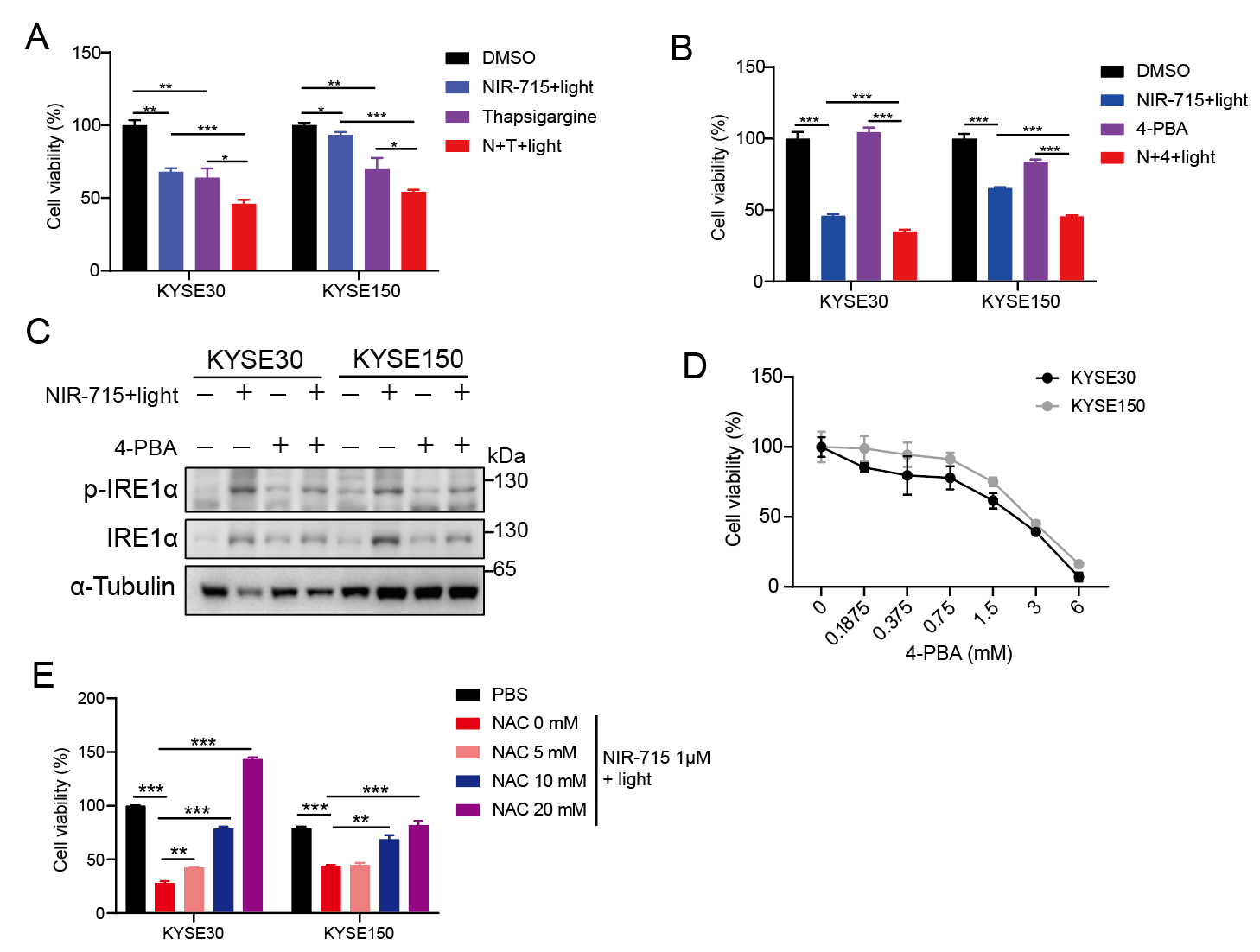
 **Figure S8** (A) Cell viability of KYSE30 and KYSE150 cells treated with DMSO, NIR-715 (1μM) + light (10 min), Thapsigargine (0.5μM), and NIR-715 + light + Thapsigargine 24 h, then the cells with or without light exposure for 10 min. The cells were cultured for another 30 min. Cell viability was evaluated using the MTS assay. (B-C) KYSE30 and KYSE150 cells treated with DMSO, NIR-715 (1μM) + light (10 min), 4-PBA (4-Phenylbutyric acid, 1 mM), and NIR-715 + light + 4-PBA 24 h, then the cells with or without light exposure for 10 min. The cells were cultured for another 30 min. Cell viability was evaluated using the MTS assay (B), the phosphorylation of IRE1α was evaluated using Western blot (C). (D) KYSE30 and KYSE150 cells treated with indicated concentration of 4-PBA 24 h, cell viability evaluated by MTS assay. (E) KYSE30 and KYSE150 cells treated with PBS, NIR-715 (1μM), NAC (5mM) + NIR-715 (1μM), NAC (10mM) + NIR-715 (1μM), NAC (20mM) + NIR-715 (1μM) 24 h and light exposure for 10 min. The cells were cultured for another 30 min. Cell viability was evaluated using the MTS assay. All data are presented as the mean ± SD (n = 4). ***P* < 0.01 ****P* < 0.001.

**
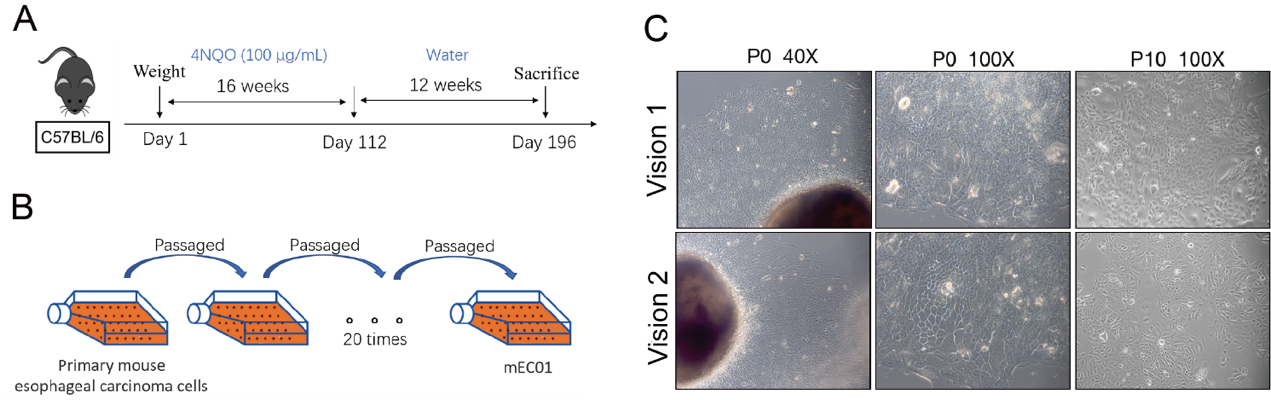
Figure S9** Establishment of the mouse mEC01-3 EC cell line. (A) Schematic illustration of 4-nitroquinoline-1-oxide (4-NQO)-induced primary esophageal cancer (EC). (B) Primary EC cells consecutively passaged for 20 generations in DMEM containing 10% FBS. (C) Cell morphology was observed by inverted microscopy (Zeiss, Oberkochen, Germany).
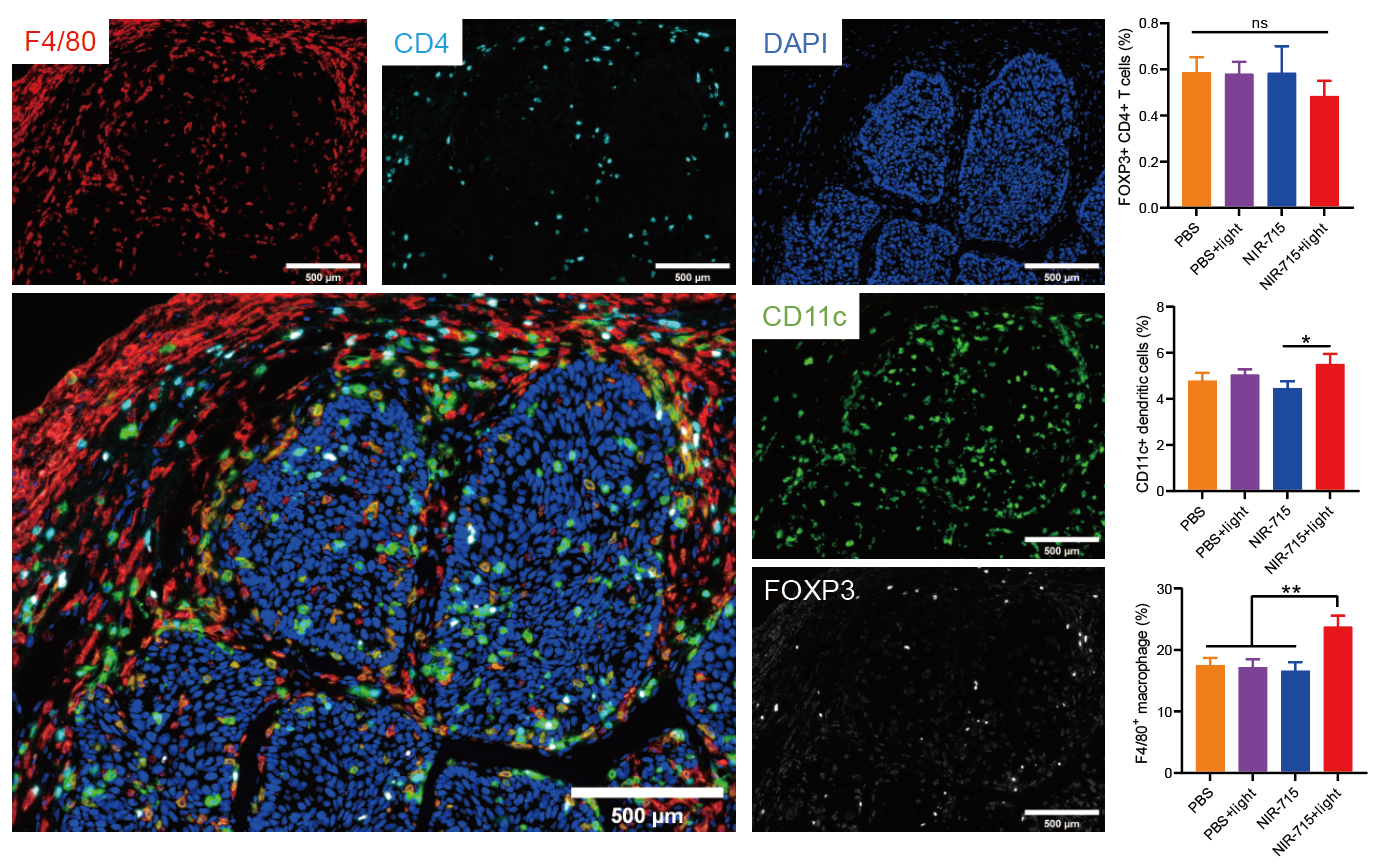


**Figure S10** Representative multiplex immunofluorescent staining image showing distribution of CD4, FOXP3, CD11c and F4/80 expression cells in mEC01-3 syngeneic mouse tumor model. Proportions of Treg cells, CD11c+ dendritic cells and F4/80 macrophage cells in mEC01-3 syngeneic mouse tumor model were examined on day 27 after NIR-715 PDT treatment. Data represent mean ± SEM (n = 5). *ns*. represents *P* > 0.05, **P* < 0.05, ***P* < 0.01. Scale bar: 500 μm.

**
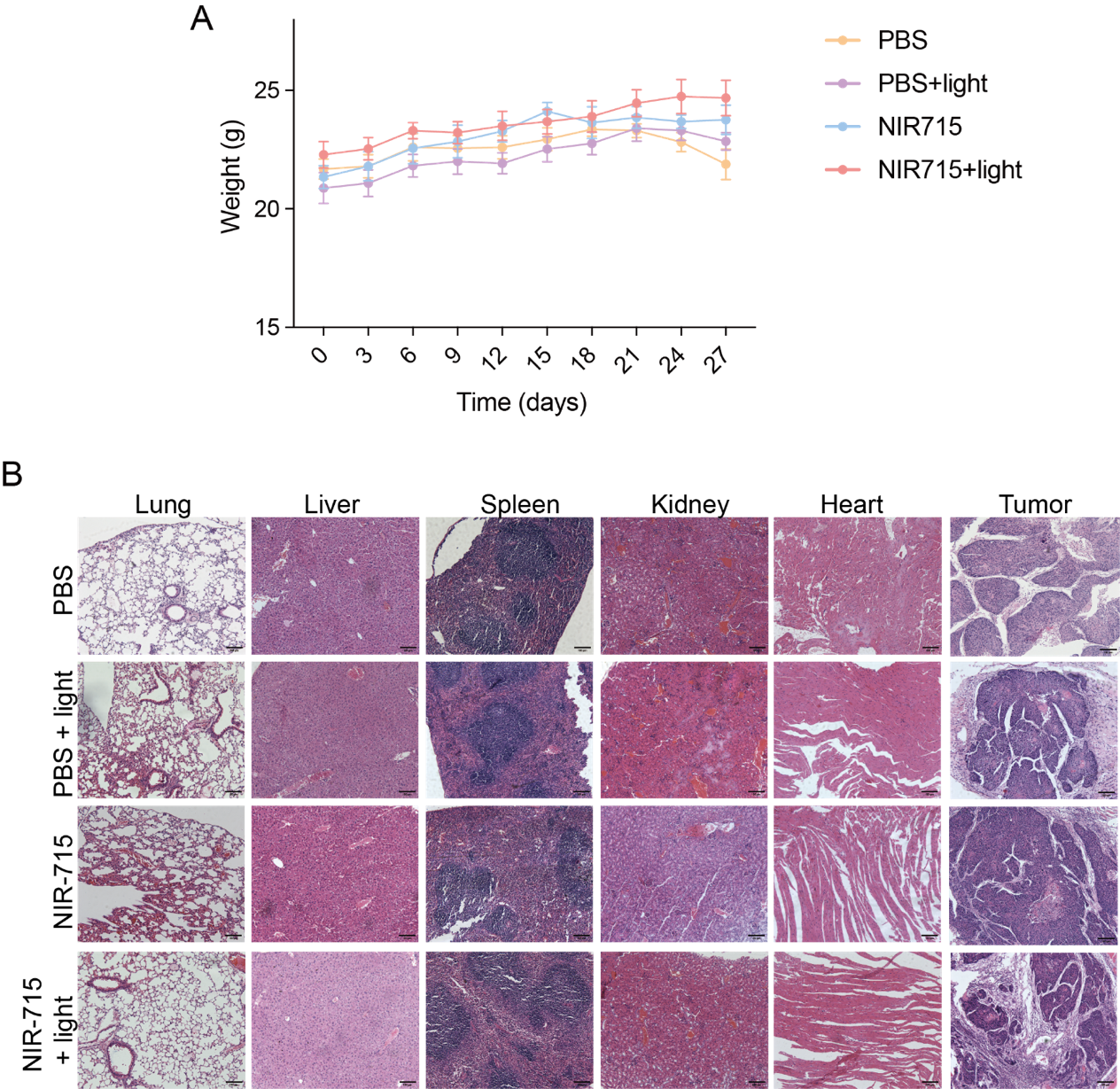
Figure** **S11** (A) Body weights of mEC01-3-bearing C57BL/6 mice. Data are presented as mean ± SEM (n = 5). (B) Representative histopathology images of the main organs and tumor. Scale bar: 100 μm.

**
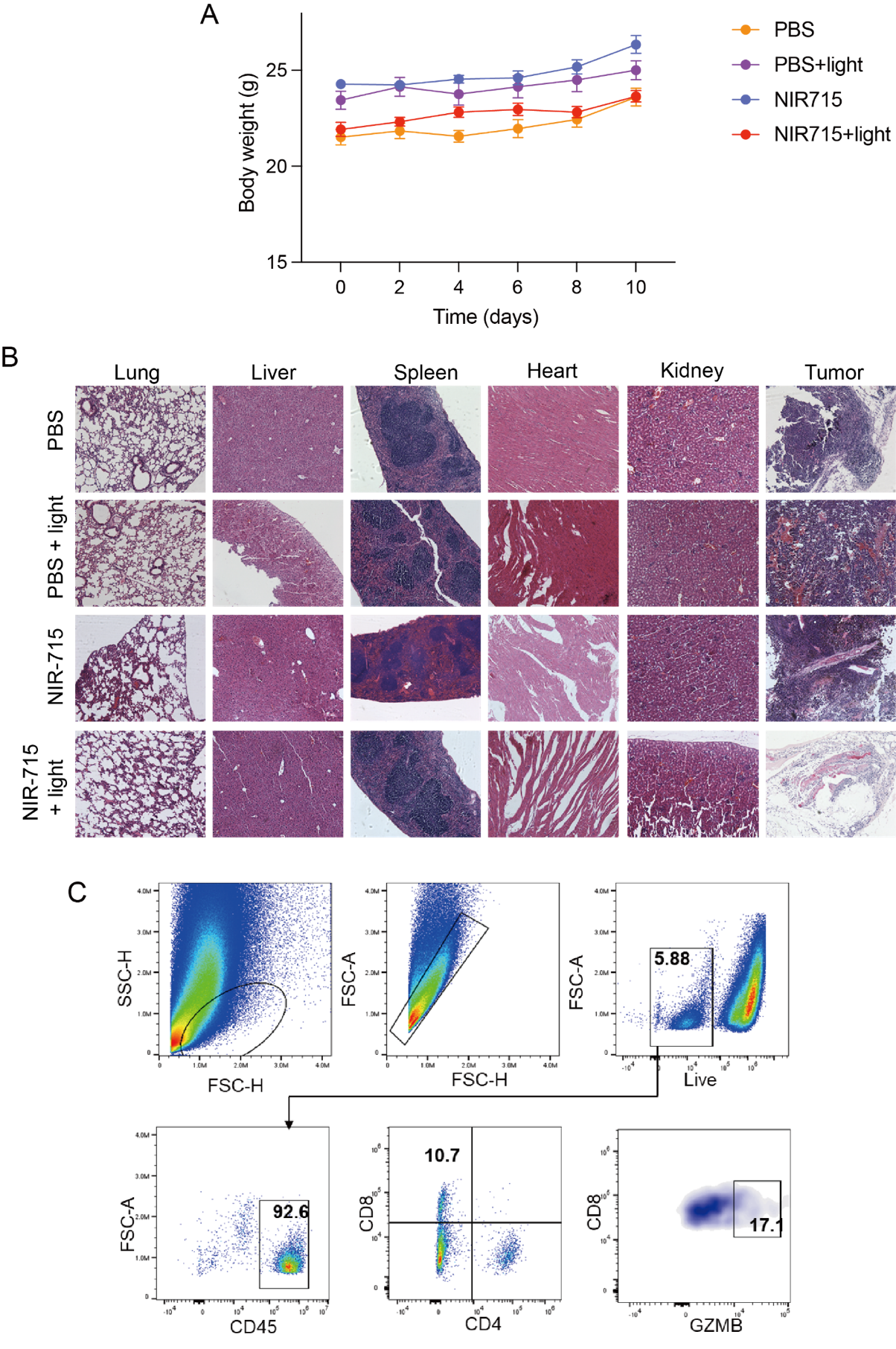
Figure S12** (A) Body weights of B16F10-bearing C57BL/6 mice. Data are presented as mean ± SEM (n = 5). (B) Representative histopathology images of the main organs and tumor. Scale bar: 100 μm. (C) Gating strategies to sort GZMB^+^ CD8^+^ T cells from B16F10-bearing C57BL/6.


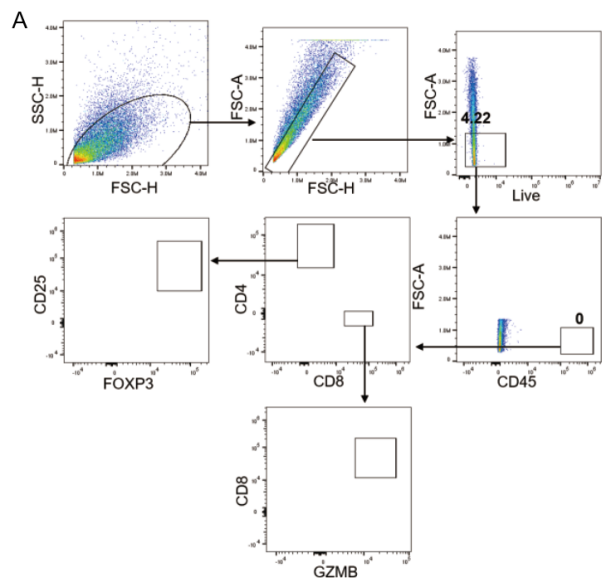

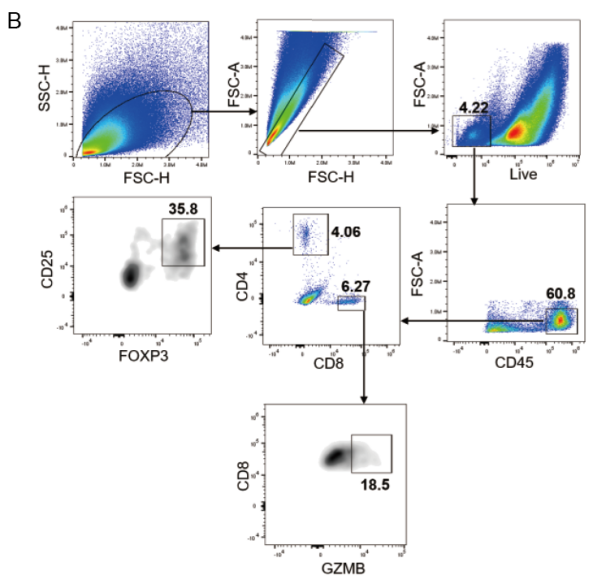


**Figure S13** Gating strategies used for cell sorting. (A-B) Gating strategy to sort CD8^+^ (CD45^+^CD8^+^) T cells, CD4^+^ (CD45^+^CD4^+^) T cells, Treg (CD4^+^CD25^+^ Foxp3^+^) cells, and GZMB^+^ T (CD45^+^CD8^+^GZMB^+^) cells from C57BL/6 mice presented on Fig. 5. A is a blank control.


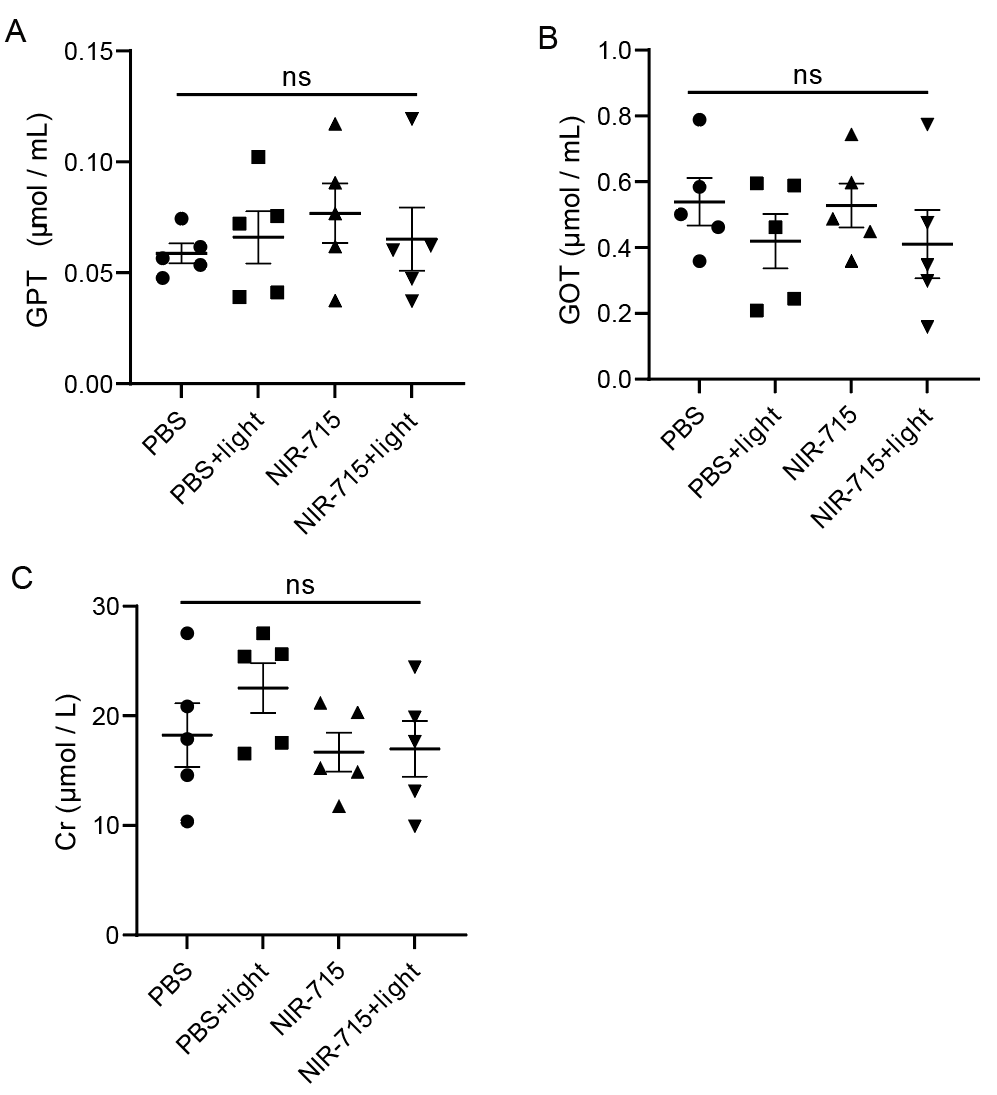
**Figure S14** Mice blood from different treatment groups was collected and centrifuged to harvest plasma, and then tested with indicated kit. GPT, glutamic pyruvic transaminase; GOT, glutamic oxaloacetic transaminase; Cr, Creatinine; *ns*. means no significant. Data are presented as mean ± SEM (n = 5).

**Video 1**: Fluorescence intensity images of KYSE150 for 24 h after NIR-715 addition

**Video 2**: Fluorescence images of ER morphology in KYSE150 cells after treatment with NIR-715.

**Video 3**: Fluorescence images of NIR-715 in KYSE150 cells after light treatment.

| **Supplementary Table 1: List of primer used in this study** | |
| --- | --- |
| **Name** | **Sequence (5'-3')** |
| hXBP1-F | GAGTTAAGAACACGCTTGGGAATGG |
| hXBP1-R | CTGATGAGGTCCCCACTGACAGA |
| CHOP-F | agctggaagcctggtatgag |
| CHOP-R | ctctgggaggtgcttgtgac |
| GADD34-F | gactgcaaaggcggctcaag |
| GADD34-R | tgcccagacagccaggaaat |
| ATF4-F | atgggttctccagcgacaag |
| ATF4-R | tctggcatggtttccaggtc |
| ACTB-F | TCCCTGGAGAAGAGCTACGA |
| ACTB-R | CTGTGTTGGCGTACAGGTCT |
