# Supplementary material for: NIR-715 photodynamic therapy induces immunogenic cancer cell death by enhancing the endoplasmic reticulum stress response
Source: Cell Death Dis. 2024 Dec 18;15(12):890. doi: 10.1038/s41419-024-07283-4 (PMC11655639; doi:10.1038/s41419-024-07283-4)
Supplement: Supplementary file 2 — uncropped original western blots. [file 41419_2024_7283_MOESM2_ESM.pptx]

## Slide 1
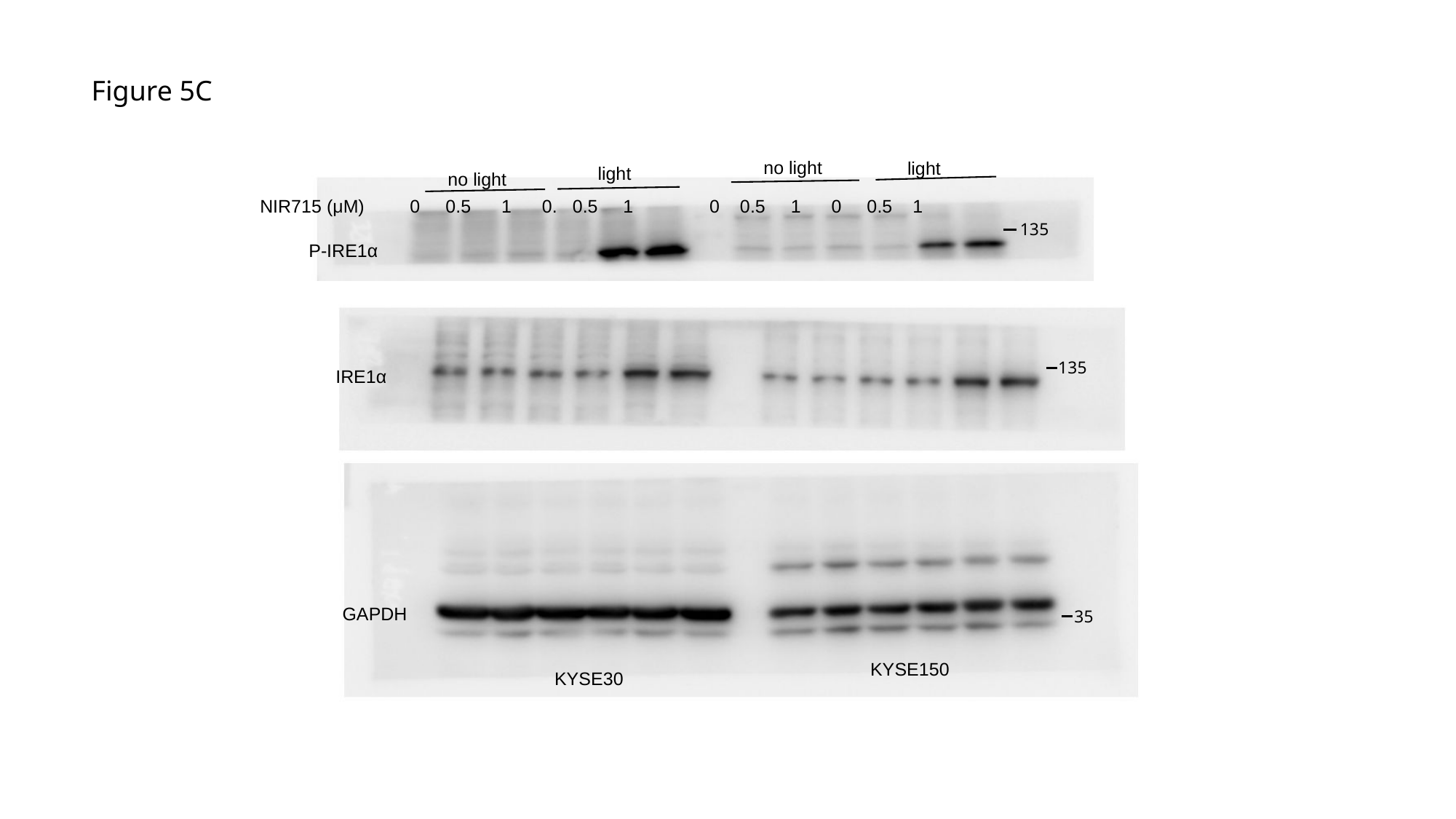

Figure 5C
no light
 light
 light
no light
NIR715 (μM) 0 0.5 1 0. 0.5 1 0 0.5 1 0 0.5 1
135
P-IRE1α
135
IRE1α
GAPDH
35
KYSE150
KYSE30

## Slide 2
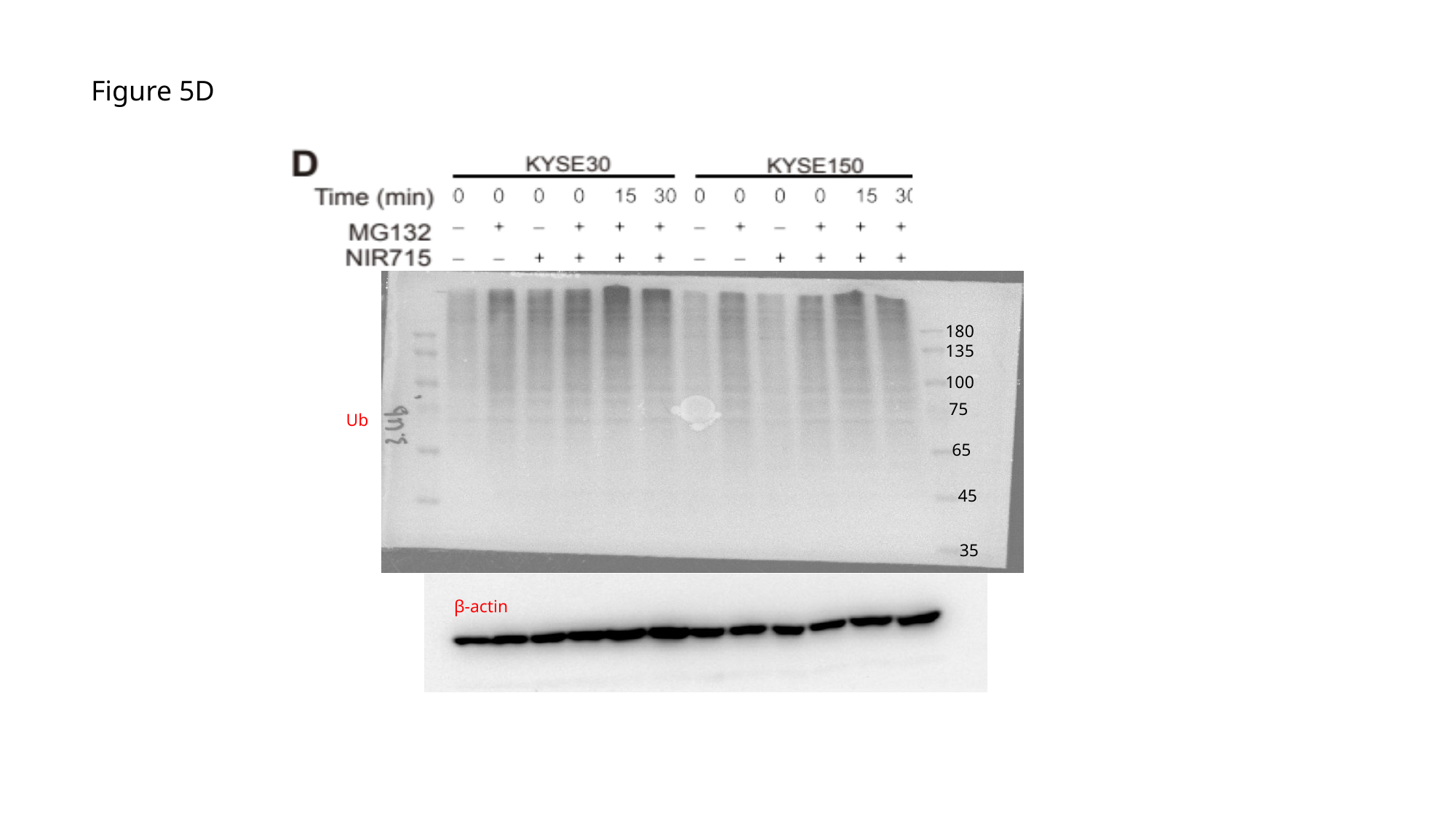

Figure 5D
180
135
100
75
Ub
65
45
35
β-actin

## Slide 3
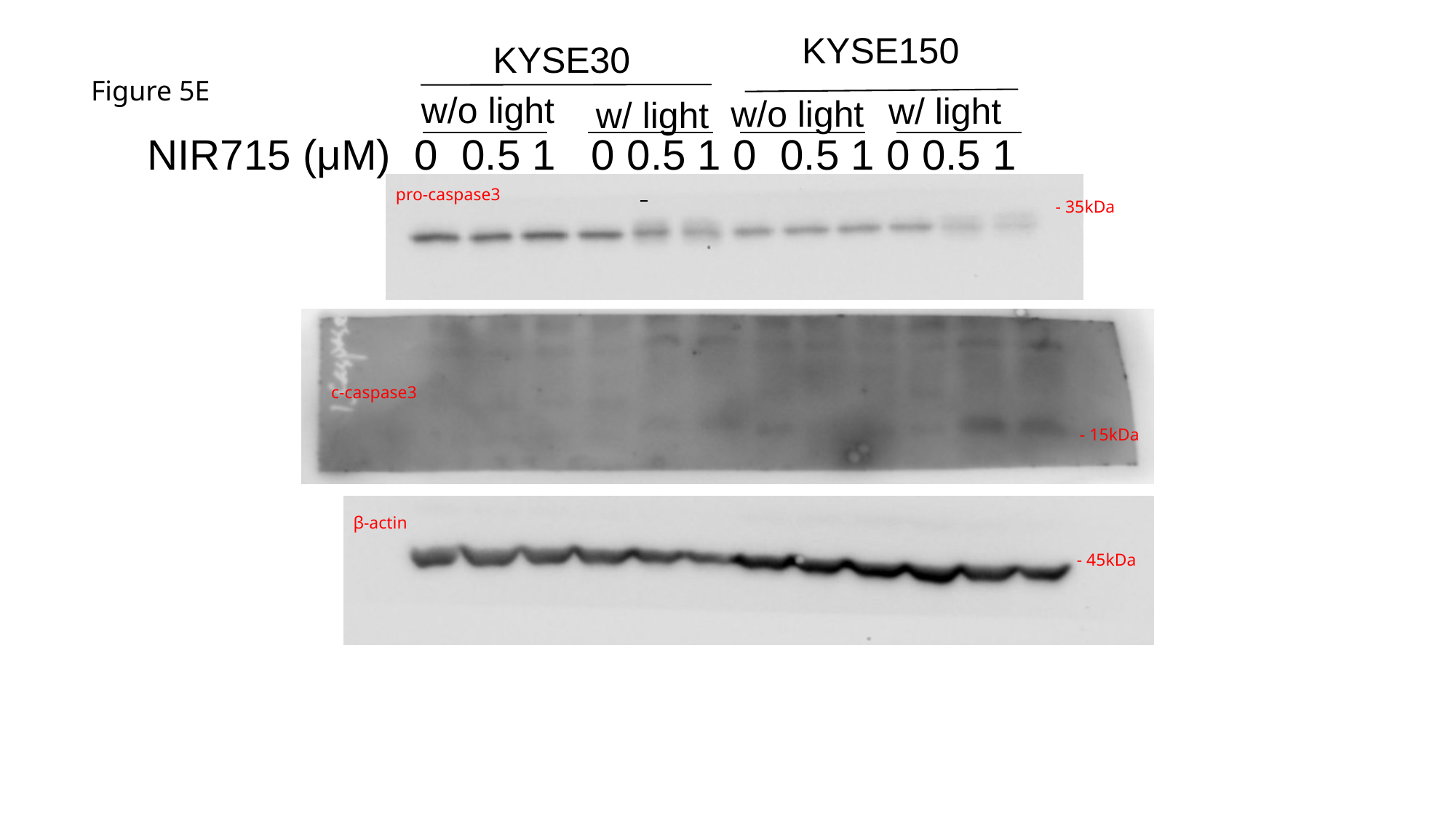

KYSE150
KYSE30
Figure 5E
w/o light
w/ light
w/o light
w/ light
NIR715 (μM) 0 0.5 1 0 0.5 1 0 0.5 1 0 0.5 1
pro-caspase3
- 35kDa
c-caspase3
- 15kDa
β-actin
- 45kDa

## Slide 4
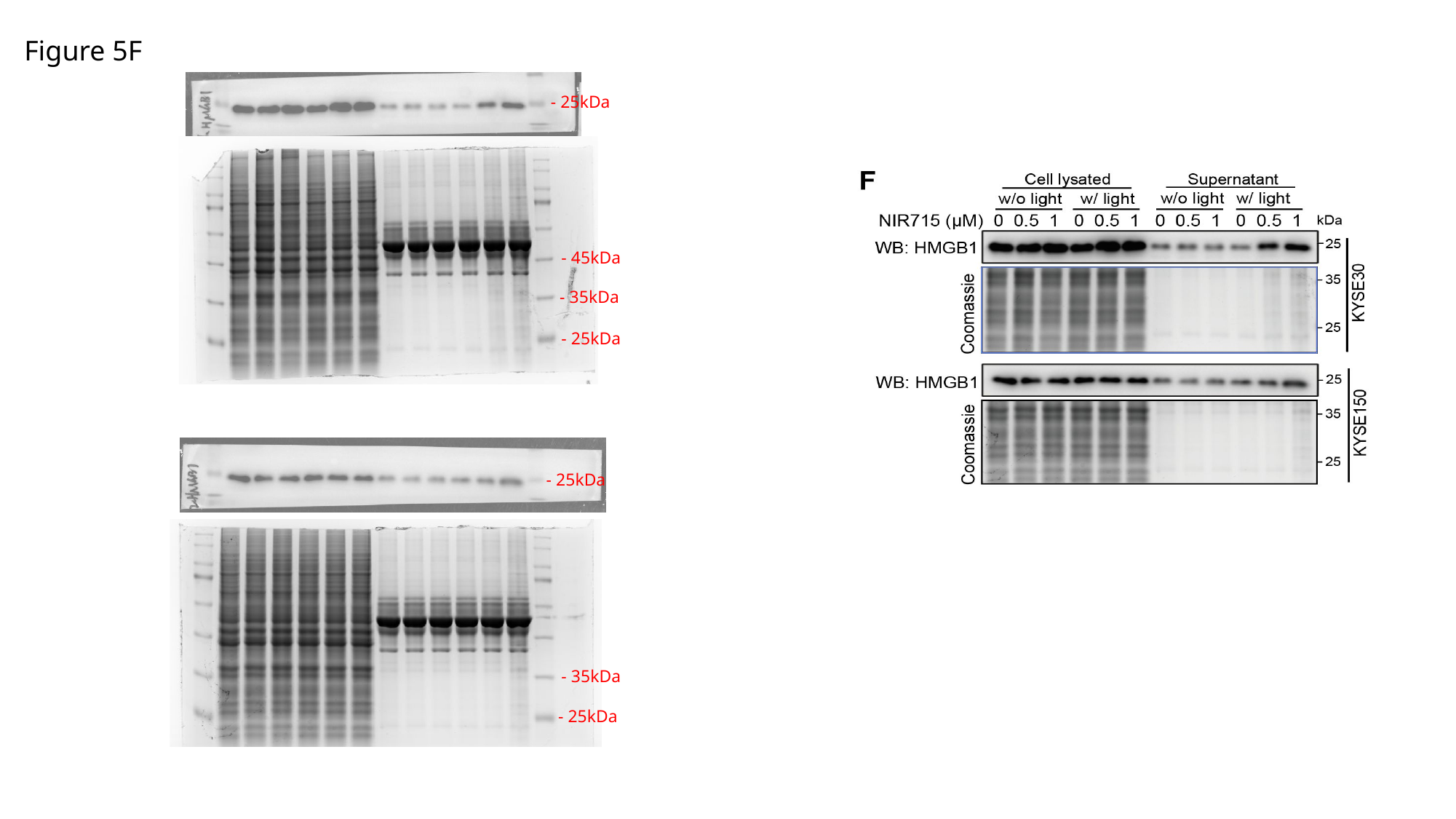

Figure 5F
- 25kDa
- 45kDa
- 35kDa
- 25kDa
- 25kDa
- 35kDa
- 25kDa

## Slide 5
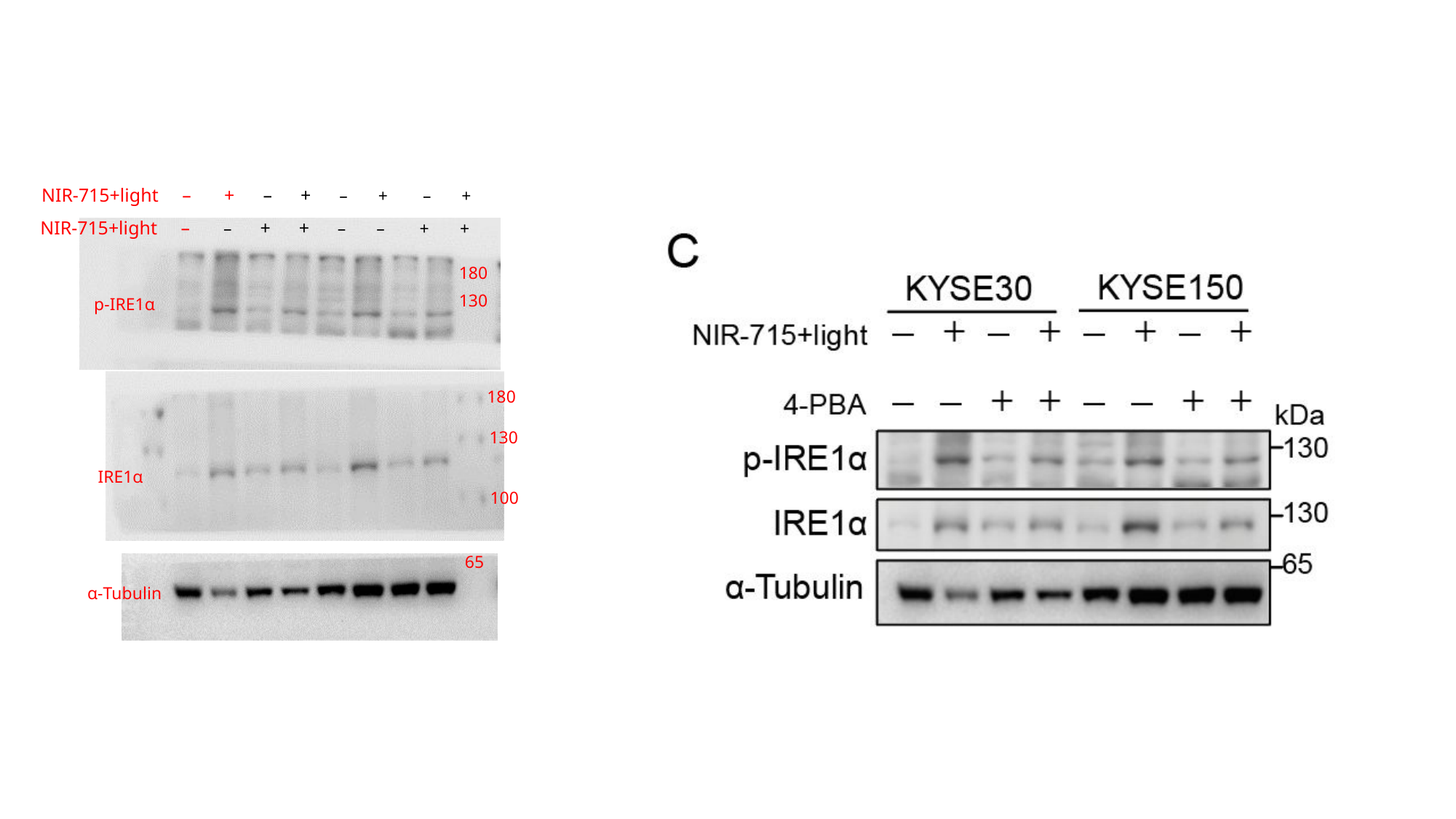

NIR-715+light – + – + – + – +
NIR-715+light – – + + – – + +
180
130
p-IRE1α
180
130
IRE1α
100
65
α-Tubulin
